# Systematic Development and Optimization of Inhalable Pirfenidone Liposomes for Non-Small Cell Lung Cancer Treatment

**DOI:** 10.3390/pharmaceutics12030206

**Published:** 2020-02-28

**Authors:** Vineela Parvathaneni, Nishant S. Kulkarni, Snehal K. Shukla, Pamela T. Farrales, Nitesh K. Kunda, Aaron Muth, Vivek Gupta

**Affiliations:** Department of Pharmaceutical Sciences, College of Pharmacy and Health Sciences, St. John’s University, Queens, NY 11439, USA; vineela.parvathaneni16@my.stjohns.edu (V.P.); nishant.kulkarni16@my.stjohns.edu (N.S.K.); snehal.shukla16@my.stjohns.edu (S.K.S.); pamela.farrales17@my.stjohns.edu (P.T.F.); kundan@stjohns.edu (N.K.K.); mutha@stjohns.edu (A.M.)

**Keywords:** repurposing, non-small cell lung cancer, pirfenidone, liposomes, 3D spheroid, apoptosis

## Abstract

Non-small cell lung cancer (NSCLC) is a global disorder, treatment options for which remain limited with resistance development by cancer cells and off-target events being major roadblocks for current therapies. The discovery of new drug molecules remains time-consuming, expensive, and prone to failure in safety/efficacy studies. Drug repurposing (i.e., investigating FDA-approved drug molecules for use against new indications) provides an opportunity to shorten the drug development cycle. In this project, we propose to repurpose pirfenidone (PFD), an anti-fibrotic drug, for NSCLC treatment by encapsulation in a cationic liposomal carrier. Liposomal formulations were optimized and evaluated for their physicochemical properties, in-vitro aerosol deposition behavior, cellular internalization capability, and therapeutic potential against NSCLC cell lines in-vitro and ex-vivo. Anti-cancer activity of PFD-loaded liposomes and molecular mechanistic efficacy was determined through colony formation (1.5- to 2-fold reduction in colony growth compared to PFD treatment in H4006, A549 cell lines, respectively), cell migration, apoptosis and angiogenesis assays. Ex-vivo studies using 3D tumor spheroid models revealed superior efficacy of PFD-loaded liposomes against NSCLC, as compared to plain PFD. Hence, the potential of inhalable liposome-loaded pirfenidone in NSCLC treatment has been established in-vitro and ex-vivo, where further studies are required to determine their efficacy through in vivo preclinical studies followed by clinical studies.

## 1. Introduction

Lung cancer is globally the leading cause of all cancer deaths among both men and women. In 2018, there were >200,000 newly diagnosed cases of lung cancer, with an estimated >142,000 deaths [1]. Lung cancer is known to be significantly more prevalent in current/former smokers and is responsible for 24% of all cancer deaths [2]. It can be categorized into non-small cell lung cancer (NSCLC) and small cell lung cancer (SCLC), out of which NSCLC has significantly higher prevalence among patients, accounting for nearly 85% of all lung carcinoma cases [3,4].

Existing first-line NSCLC treatment options include surgical resection, radiation therapy, chemotherapy [5,6], and targeted therapies (NSCLC with driver oncogene mutations) or immune checkpoint therapies [7]. However, patient outcomes still remain discouraging due to either poor/late diagnosis, lack of first-line chemotherapeutics, and/or lower survival rates after surgical treatment [8,9]. Moreover, chemotherapy is associated with higher systemic side effects [1,10] while also establishing resistant tumors [9,11]. This is a significant barrier for effective NSCLC treatment and is responsible for the majority of tumor metastases and reoccurrence [12]. While it is crucially important to discover and commercialize novel and more effective chemotherapeutic agents, slow progress in new drug development hampers scientific advancements. For a molecule to be approved by the FDA, a potential drug candidate has to undergo in-vitro/in-vivo/clinical safety and efficacy studies, which would take approximately 10 to 14 years. Drug repurposing (i.e., investigating old drugs for their new therapeutic use) presents an attractive alternative to significantly shorten this period to 4 to 5 years while reducing drug development costs [13,14]. In this milieu, drug repurposing provides an opportunity to identify a new indication for an old FDA-approved drug [13,15].

Pirfenidone ((5-methyl-1-phenylpyridin-2(1H)-one)) (PFD) is an anti-fibrotic drug that was approved by the FDA for treatment of idiopathic pulmonary fibrosis in 2014 (Esbriet^®^). Due to commonalities in fibrotic, cancer proliferative, and pathogenic mechanisms, PFD was evaluated for and found to exhibit some anticancer properties, as reported by Mediavilla-varela et al. and Fujiwara et al. [16,17]. Other researchers have explored the importance of PFD in combination with other chemotherapeutic agents, such as carboplatin, to cause tumor cell death in NSCLC [16]. Recently, Usugi et al. demonstrated the anti-proliferative action of PFD on pancreatic cancer cells in-vitro [18]. Hence, it is evident that a PFD has the potential to be repurposed as an anti-cancer drug. However, PFD’s utility as an anti-cancer therapy may be hindered by systemic adverse effects such as photosensitivity, anorexia and nausea, which are often a result of a higher dose requirement and off-target accumulation of PFD [19]. Hence, localized and site-specific delivery of PFD to tumor site will be instrumental in establishing it as an anti-cancer drug of choice.

As primary NSCLC tumors are accumulated in deep lung regions, it will be imperative to develop an inhalable delivery system for localizing the therapy at the location of occurrence. While considering delivery to the respiratory system, it is of paramount importance to appreciate the lipid-rich environment of the lungs. Liposomes (i.e., the unilamellar lipid vesicles) have been extensively explored for their ability to carry both hydrophobic and hydrophilic drugs deep inside the lungs [20]. Moreover, inhalable liposomal formulations are proven for their ability to deliver the drugs locally and to facilitate targeted delivery in other lung disorders [20,21]. For instance, Amikacin liposomes (ARIKAYCE^®^) have been approved by the U.S. Food and Drug Administration for inhalation therapy via nebulization in treating Mycobacterium avium complex (MAC) [22]. Liposome formulations are required to be aerosolized into particles with optimal aerosolization properties and liposomes should also encapsulate a therapeutically feasible amount of drug, which can then exhibit prolonged release from the liposome vesicles to the target site within the lung. Moreover, inhaled drugs may be trapped within the mucus and subsequently removed with multiple clearance mechanisms. Hence, it is required to manipulate the physicochemical properties of PFD, such as surface charge, and hydrophobicity to avoid the entrapment of drugs in the mucus and increase the penetration of drugs across the mucus layers [23]. By varying lipid compositions, surface charge, and membrane fluidity, higher payload capacities can be achieved [24]. As we propose to evaluate the efficacy of PFD as a lung cancer therapeutic, the delivery of PFD through a liposomal drug delivery system is explored in this study. Liposomes can aid in drug accumulation at the target site, thereby reducing systemic toxicity. As the major mechanism of efficacy enhancement for liposomes is to promote increased drug accumulation at disease site/cells, we propose to explore the incorporation of a cationic lipid, 1,2-dioleoyl-3-trimethylammonium-propane (chloride salt) (DOTAP), in the lipid film. This will improve cellular uptake of PFD-loaded liposomes, owing to positive surface charge and favorable interaction with the negatively charged cell membrane, as reported by Baczynska et al. [25]. A freeze–thaw technique is exploited in this study to achieve higher PFD loading in liposomes [26]. The objective of this study is to establish the efficacy of cationic liposomes loaded with PFD for NSCLC treatment in a bid to provide higher therapeutic and socioeconomic value than current NSCLC treatment regimens.

## 2. Materials and Methods

### 2.1. Materials

1,2-dipalmitoyl-*sn*-glycero-3-phosphocholine (DPPC), cholesterol and 1,2-dioleoyl-3-trimethylammonium-propane (chloride salt) (DOTAP) were procured from Avanti polar lipids (Alabaster, AL, USA). Pirfenidone (5-methyl-1-phenylpyridin-2(1H)-one) was obtained from Oakwood chemicals (Estill, SC, USA). A549, H4006, H460, and H157 NSCLC cell lines were obtained from ATCC and maintained in RPMI medium (Corning) supplemented with 10% FBS (Atlanta Biologicals, Flowery branch, GA, USA), sodium pyruvate, penicillin-streptomycin (Corning) at 5% CO_2_/37 °C. 3-(4,5-dimethylthiazol-2-yl)-2,5-diphenyltetrazolium bromide (MTT), coumarin-6, crystal violet dye, 16% paraformaldehyde, HPLC grade methanol, acetonitrile (ACN), and water were all purchased from Fisher Scientific (Hampton, NH, USA). Molecular biology kits, supplies, and antibodies were purchased from other commercial vendors, which are listed at appropriate places throughout the manuscript.

### 2.2. Methods

#### 2.2.1. UPLC Method Development for Pirfenidone (PFD)

A reverse-phase liquid chromatography method was developed for quantifying pirfenidone using a Waters series Acquity UPLC (Waters, Milford, MA, USA) using an Xselect^®^ CSH C18 (3.0 × 50 mm; 2.5 μm particles) column. The mobile phase consisted of HPLC grade water at pH 4.5 (adjusted using orthophosphoric acid, 85%) and organic phase (methanol:ACN 45:55) at 60:40 at a flow rate of 0.4 mL/min at 210 nm wavelength. Retention time was found to be 1.73 min with a total run time of 4 min. Data were collected and analyzed using Empower 3.0 software.

#### 2.2.2. Formulation and Optimization of Pirfenidone Liposomes

The composition of all PFD-loaded liposomal formulations is presented in Table 1. Formulations F1-F4 were prepared using the thin-film hydration technique followed by either passive (F1, F2) or active (F3, F4) loading of PFD, as reported previously [27]. Briefly, a thin lipid film of 20 mM DPPC and cholesterol (molar ratio of 7:3 or 5:5) was formed by subjecting lipid solution in chloroform to evaporation using a rotary evaporator (Wilmad-LabGlass, Vineland, NJ, USA) for 2.5 h under reduced pressure of 200 bar at 40 °C. PFD (5 mg) was loaded either passively through hydrating with aqueous drug solution (F1, F2); or through pH-dependent active loading (250 mM ammonium sulfate solution, pH 3.0; F3, F4) followed by sequential extrusion through Whatman nucleopore membranes (0.8, 0.2, 0.1 µm), each for 21 passages, using Avanti’s mini extruder (Alabaster, AL, USA). Extruded PFD-loaded formulations were separated from free PFD by size exclusion using a Sephadex^TM^ G-25 M column equilibrated with PBS (1X, pH 7.4). In the case of active loading, lipid film was hydrated with pH 3.0 solution consisting of 250 mM ammonium sulfate. Following hydration, the outside pH was changed to 7.0 using a Sephadex G25M column, and liposomes were extruded, as described earlier. Liposomes after extrusion were incubated with PFD at 65 °C for 60 min followed by column separation as described earlier. In regards to F5–F7, passive loading approach was used to load PFD (5 mg: F5, F6; 10 mg: F7) where drug was loaded inside the lipid film followed by hydration with 10 mM HEPES ((4-(2-hydroxyethyl)-1-piperazineethanesulfonic acid) buffer (pH 7.4 in 140 mM sodium chloride) and ultra-probe sonication (4 min at 40% amplitude; 10 s on-off cycles) was carried out for size reduction of liposomes using method reported by Meng and Xu [28]; and Togami et al. [29] with slight modifications. F6 and F7 were further subjected to two freeze–thaw cycles by freezing the liposomes using liquid nitrogen at −196 °C for 3 min, followed by thawing in a water bath at 45 °C for 3 min. To further increase the drug loading, F8, F9, and F10 were formulated using a similar approach as used for F6 and F7. Here, the 10 mM lipid film consists of DPPC (36.7, 34.86, or 33.03 mg), cholesterol (19.3 mg) and PFD (20 mg), with or without DOTAP (DOTAP concentration (% *w/w* of total lipid))—(0%, 5%, and 10% for F8, F9, and F10, respectively), as seen in Table 1**.** F8 and F9 were named as PFD–Lip and PFD–D-Lip, respectively, and were used in further studies.

#### 2.2.3. Characterization of Liposomes

##### Particle Size, Poly Dispersity Index (PDI), Zeta Potential, and Phospholipid Quantification

Particle size, PDI, and zeta potential were determined by dynamic light scattering (DLS) using a Zeta Sizer (Malvern Instruments, Malvern, UK). The formulations were diluted 100-fold with milli-Q water to obtain samples for sizing and zeta potential measurements at room temperature. The respective samples were loaded into the zeta cells (DTS 1070) and analyzed. Phospholipid content of the PFD–Lip and PFD–D-Lip was quantified using a phospholipid assay kit (MAK122, Sigma Aldrich, MO, USA). Briefly, the standard curve of phospholipid was plotted using a colorimetric assay, as per the manufacturer’s instructions. Liposomal formulations were lysed with organic solvent, as described in Section 2.2.1. The supernatants were further diluted with milli-Q water, transferred to 96-well plates, and further steps were carried out according to the protocol, and absorbance was determined on a Spark 10M (Tecan, Männedorf, Switzerland) plate reader at 570 nm.

##### Drug Content

To determine the amount of drug loaded on the liposomal formulation, a direct vesicle lysis approach was followed. To 20 μL of liposomes, 1980 µL of a mixture of methanol:ACN (45:55) was added followed by centrifugation at 21,000× *g* for 45 min (4 °C) to lyse the liposomes and to release the loaded drug into analyzing solution. Clear supernatant obtained was collected, analyzed for the drug content using the UPLC method, as described earlier. Then, % entrapment efficiency (EE%) and % drug loading were calculated using the below equations.

$$\%\;EE=\left[\frac{Entrapped\;drug}{Total\;drug\;taken\;initially}\right]\times 100$$

$$\%\;Drug\;Loading=\left[\frac{Entrapped\;drug}{Total\;lipid+drug\;amount}\right]\times 100$$

##### In-Vitro Release Studies

In-vitro release studies were performed for determining the drug release pattern from PFD–Lip and PFD–D-Lip using the dialysis method, as reported previously [27]. Briefly, 2,000 MWCO dialysis cassettes (Slide-A-Lyzer, 0.2–0.5 mL, Thermo-Scientific, Waltham, MA, USA) were preconditioned in simulated lung fluid buffer (pH 7.4) for 10 min to allow the cassettes to become hydrated. Simulated lung fluid (SLF-3) was prepared according to the literature, and to account for the lung surfactant present, DPPC was added to that in the form of liposomes [30]. DPPC liposomes were prepared by thin film hydration technique by dissolving 200 mg of DPPC in 40 mL of chloroform: methanol mixture (1:1) and subjecting for rotary evaporation to obtain thin lipid film. Then the film was hydrated with 200 mL of water and agitated for 2 h at 55 °C followed by sonication for 1 h at 55 °C. These concentrated DPPC liposomes were added to SLF-3 and the pH of this dissolution medium was maintained at pH 7.4 using 0.5 M sodium phosphate monobasic. For release studies, 500 µL of the formulation was loaded into the cassette membrane though one of the ports using a syringe with a 19G1_1/2_ TW BD filter needle. Later, the cassettes were immersed in a beaker containing 150 mL of SLF-3 with DPPC liposomes (pH 7.4; 37 °C) using a floater while stirring at 200 rpm speed. At regular time points of 0.5, 1, 2, 4, 6, 8, 12, 24, and 48 h, samples were withdrawn while replenishing the dissolution medium with fresh SLF-3 with DPPC liposomes. The amount of drug released was estimated using the UPLC method, as discussed above.

##### Morphological Analysis Using Transmission Electron Microscopy (TEM)

Morphology of liposomal formulations was studied by transmission electron microscope (TEM). Briefly, 20 μL drops of each sample solution (PFD–Lip or PFD–D-Lip) was displayed on a parafilm sheet. Then, formvar-carbon-coated copper grids (100 mesh, Electron Microscopy Sciences, Hatfield, PA, USA) were placed on it. After allowing the material to adhere to the grids for 10 min, grids were washed 3 times by rinsing through 200 μL drops of Milli-Q water before being left for 1 min on 2% *w/v* uranyl acetate (Ladd Research Industries, Williston, VT, USA). Excess solution was removed with Whatman 3MM blotting paper, and grids were left to dry for a few minutes before viewing. Grids were examined using a JEOL JEM-1400 Plus transmission electron microscope operating at 80 kV. Images were recorded using a Gatan OneView 4K digital camera (Gatan Inc., Pleasanton, CA, USA).

##### Solid State Characterization Studies

These studies were carried out by using the powder form of PFD–D-Lip obtained through the freeze-drying of liposomal formulations.

Powder X-ray Diffraction (PXRD) Studies: X-ray diffraction spectroscopy was carried out using XRD-6000 (Shimadzu, Kyoto, Japan). The diffractometry was performed by using a graphite monochromator consisting of copper-Kα1 radiation of wavelength 1.5418 Å operating at 40 kV, 30 mA. The samples were spread uniformly on a glass micro-sample holder and were analyzed in the range of 10° to 60° at the scanning speed of 2° (2θ)/minute.

Differential Scanning Calorimetry (DSC) Studies: Thermograms for PFD, PFD–D-Lip, blank D-Lip, and physical mixture of PFD and blank D-Lip were generated using a DSC 6000 (PerkinElmer; CT, USA) equipped with an intra-cooler accessory. An accurately weighed sample (1–5 mg) was sealed in an aluminum pan and analyzed over a temperature range of 30 to 210 °C and compared to a sealed empty aluminum pan maintained as a reference. The heating rate was maintained at 10 °C/min under a nitrogen purge with a flow rate of 50 mL/min.

In-Vitro Aerosol Performance Lung Deposition Test: In-vitro lung deposition behavior of PFD–D-Lip was evaluated using an M170 Next Generation Impactor™ (NGI: MSP Corporation Shoreview, MN, USA) in accordance with earlier published studies [31]. Briefly, the NGI was equipped with a stainless-steel induction port (USP throat adaptor) and insert cups. PFD–D-Lip formulation (2 mL) was placed into a PARI LC PLUS^®^ nebulizer cup of a Pari FAST-NEB compressor system (Boehringer Ingelheim Pharmaceuticals, Inc. Ridgefield, CT, USA) and attached to a customized rubber mouthpiece connected to the NGI. The flow rate was adjusted to 15 L/min using an HCP5 vacuum pump (Copley Scientific, UK) and a DFM 2000 flow meter (Copley Scientific, NG4 2JY, UK). Then, 2 mL of the formulation was nebulized with a PARI LC PLUS^®^ nebulizer, which passed through induction port into the NGI using a pump at a flow rate of 15 L/min for 4 min. Prior to running the NGI, the plates were refrigerated at 4 °C for 90 min to cool the NGI plates. Samples were collected from each stage, i.e., Stages 1–8, including throat and induction port as well, which is important in determining the emitted dose through rinsing with methanol:ACN (45:55) and analyzed by UPLC for drug content and deposition, as discussed above. All experiments were performed in triplicate (*n* = 3). Fine particle fraction (FPF, %) was determined as the fraction of the emitted dose deposited in the NGI with d_ae_ < 5.39 µm. Mass median aerodynamic diameter (MMAD, D_50%_) and geometric standard deviation (GSD) are the critical parameters for inhalation testing and were calculated by quantifying the liposomal deposition at each stage in the NGI using log probability analysis (*n* = 3) [31,32].

#### 2.2.4. Cellular Uptake Studies

Cellular uptake studies were performed using a protocol reported earlier [33]. Briefly, A549 cells were plated in tissue culture (TC) treated cell imaging cover-glass (8 chambers; Eppendorf, Hauppauge, NY, USA) at a seeding density of 10,000 cells/chamber followed by overnight incubation. The next day, cells were incubated with coumarin-6, coumarin-6 loaded Lip, or D-Lip at 1 µg/mL concentration for 1, 3, or 24 h. After each time interval, cells were washed with ice-cold, sterile PBS twice and fixed with 4% PFA for 10 min. Fixed cells were twice washed again with ice-cold PBS, chambers were removed, and 20 µL of Vectashield hardset mount with DAPI nuclear stain (H1500, Vector laboratories, Burlingame, CA, USA) was placed on a glass slide dropwise followed by placing of the inverted cover glass on it. After the hardening of mounting medium overnight at 4 °C, the slide was imaged using an EVOS-FL microscope (Thermo Scientific, Waltham, MA, USA).

#### 2.2.5. Stability Studies

The stability of the liposomal formulations F8 (PFD–Lip) and F9 (PFD–D-Lip) was evaluated while storing the samples at temperatures of 4, 25, and 37 °C for four (4) weeks, as previously reported [27]. Samples were withdrawn after weeks 1, 2, 3, and 4, diluted with water 100-fold, and analyzed for particle size, PDI, and zeta potential using a Malvern Zeta Sizer. Entrapment efficiency was determined by lysing the samples, as described earlier, using UPLC. All these experiments were performed in triplicate (*n* = 3).

#### 2.2.6. Cytotoxicity Studies

PFD–Lip and PFD–D-Lip, along with plain PFD, were evaluated for their cytotoxic potential in four different non-small cell lung cancer cell lines: A549, H157, H460, and H4006. Briefly, cells were grown in FBS supplemented RPMI-1640 media as described in the Materials section, and were seeded in TC treated 96-well plates (Eppendorf, Hauppauge, NY, USA) at a seeding density of 2500 cells/well (7500 cells/cm^2^), incubated overnight for adherence at 37 °C/5% CO_2_, and treatments were added next day at different PFD concentrations ranging from 0.0625–2.0 mg/mL. Corresponding volumes of PFD–Lip and PFD–D-Lip were calculated based on the drug entrapment efficiency. Blank culture media was added as control. After 72 h incubation, % cell viability was determined by MTT assay. In brief, the media was aspirated from the wells, and cells were incubated with 100 µL of MTT solution (1 mg/mL in sterile PBS) for 2 h. Subsequently, MTT solution was aspirated and 100 µL of DMSO was added to dissolve formazan crystals in each well. The plates were then shaken for 30 min on a plate shaker. The absorbance was read on a Spark 10M (Tecan, Männedorf, Switzerland) plate reader at 570 nm. The safety of optimized liposomal formulations was determined by evaluating the cytotoxicity of Blank–Lip and/or Blank–D-Lip on two NSCLC cell lines, A549 and H1299, and a human embryonic kidney (HEK-293) cell line. Cells were seeded into 96-well plates as described earlier and were incubated overnight. A549 and H1299 cells were treated with equivalent volumes of Blank–Lip and Blank–D-Lip to 0.1, 0.2, 0.5, and 1 mg/mL of PFD–Lip and PFD–D-Lip, respectively, and incubated for 72 h. HEK cells were treated with equivalent volumes of Blank–D-Lip to 0.1, 0.5, 1, and 2 mg/mL of PFD–D-Lip and incubated for 24 or 72 h. Then, % cell viabilities were determined through MTT assay, as described earlier.

#### 2.2.7. Scratch Assay

An in-vitro scratch assay was used to study cancer cell migration. Briefly, scratches were created on a confluent cell monolayer, causing the cells on the scratch’s edge to migrate toward the center to close the scratch, thus establishing new cell–cell contacts. The assay was performed as previously reported [34]. Briefly, A549 cells were plated in 24-well cell culture plates (1 × 10^5^ cells/well) followed by overnight incubation. The next morning, scratches were made along the center of all wells with the help of a p200 (200 µL) sterile pipette tip. All the wells were washed with sterile PBS twice and reference markings were drawn near the scratch area from the bottom side of the plate with a fine tip marker. Then, scratch images were captured within the marked area using an inverted microscope (Laxco, Mill Creek, WA, USA) with a 10X magnification objective piece. Later, treatments of control, PFD, and PFD–D-Lip (0.5 mg/mL) were added to the respective wells and incubated, followed by imaging after 24 and 48 h. Images captured were analyzed quantitatively to assess the inhibitory effect of PFD and PFD-loaded liposomes on cell migration, and scratch width using ImageJ software and % scratch closure was further calculated.

#### 2.2.8. Clonogenic Assay

Clonogenic assay is an in-vitro cell survival assay which is based on a single cell’s capability to grow into a colony. Using this assay, the effectiveness of PFD and PFD–D-Lip toward colony inhibition was determined. The protocol reported previously [35] was modified and followed in this study. From confluent flasks of A549 and H4006 cell lines, cells were seeded into 6-well cell culture plates at a seeding density of 250 cells/mL and volume of 1 mL/well. The plates were incubated overnight to allow the cells to properly adhere to the plate. The next day, media was replaced and cells were treated with PFD (0.5 mg/mL), PFD–D-Lip (0.5 mg/mL), or control (media) for 48 h. After 48 h, all treatments were removed and fresh media replacements were done on alternative days for a 7-day period. On the 7th day, the media was removed from all the wells and colonies were stained with crystal violet. Briefly, contents of the well were removed and washed with ice-cold PBS buffer twice followed by fixation with 4% PFA solution (in ice-cold PBS) for 10 min. Fixed cells were again washed with ice-cold PBS twice, followed by staining with 0.01% (*w/v*) crystal violet solution with a 1-h incubation. After staining, cells were washed with distilled water and images were captured using a digital camera. Cell colonies were counted by colony counter software Open CFU [36].

#### 2.2.9. In-vitro Angiogenesis Study

Angiogenesis is the formation of new blood vessels from pre-existing vasculature and is a key process in specific disease development. Over-proliferation of blood vessels is considered a pivotal pathogenic mechanism in NSCLC, among other cancer types [37]. Recently, PFD was suggested to demonstrate significant anti-angiogenic activity in wound healing processes. To understand PFD-loaded liposomes’ anti-angiogenic ability, an angiogenesis assay was performed as per the manufacturer’s instructions (R&D Systems; Minneapolis, MN, USA). Briefly, human umbilical vein endothelial cells (HUVEC) were seeded into a T-25 flask prior to performing an angiogenesis assay. The next day, 50 μL of Cultrex^®^ RGF BME was added to each well of a 96-well plate and incubated at 37 °C for 60 minutes to allow the BME to gel. HUVEC cells were then labeled with 2 μM calcein AM and seeded at 1.3 × 10^4^ cells/well on a BME-coated 96-well plate after preparing dilutions with varying concentrations (0.5 and 1 mg/mL) of PFD and PFD–D-Lip. Cells were incubated for 6 hours at 37 °C/5% CO_2_. Images were taken using a fluorescence microscope (EVOS FL, Thermo Scientific, Waltham, MA, USA), and tube formation was evaluated and quantified using NIH ImageJ software with the angiogenesis analyzer plug-in [38].

#### 2.2.10. 3D Spheroid Study

To understand the ability of PFD-loaded liposomes to penetrate through actual tumor mass and provide superior therapeutic efficacy, we performed 3D spheroid culture studies. 3D spheroid culture methods provide a way to evaluate a drug candidate ex-vivo while mimicking the in-vivo appearance and behavior of tumor mass. We recently described the capability of spheroid cell culture studies to mimic the in-vivo features of tumors [39]. This spheroid study was conducted utilizing two kinds of models, prophylactic and therapeutic, which differ significantly in tumor growth and treatment strategies.

In a prophylactic study, 2000 cells (A549, H460, or H4006) were seeded into each well of a Nunclon Sphera 96-well plate (Thermo Fisher Scientific, Waltham, MA, USA) and were incubated overnight under standard conditions of 37°C/5% CO_2_. The next day, media was replaced with either fresh media (control) or respective treatment solutions of PFD, PFD–D-Lip at (A549: 0.25, H460: 0.125, and H4006: 0.25 mg/mL). On day 1, all the wells were observed for spheroid formation with a rigid margin; images were captured using an inverted microscope (Laxco, Mill Creek, WA, USA). NIH ImageJ software was used to measure the diameter of all the spheroids. Spheroids were continuously observed, and images were captured on days 3, 5, 7, 10, and 15 following treatment. At each time-point, 100 µL of media was replaced with fresh culture media in a gentle manner to avoid bubble formation and aspiration of the spheroid itself.

In a therapeutic 3D model, H460 or A549 cells were seeded into a Nunclon Sphera plate at a density of 500 cells per each well and incubated at 37 °C/5% CO_2_. All wells were then observed for spheroid growth, and images were captured on days 1, 3, 5, and 7. On day 7, spheroids were subjected to a single dose and a multiple-dose treatment. Briefly, both single and multiple dosing spheroids were treated with 0.5 mg/mL concentrations of PFD and PFD–D-Lip (to maintain original concentrations employed in the beginning), and images were captured. For regular dosing, fresh media was provided on the 8th, 10th, and 13th day, and for multiple doses, wells were replenished with PFD and PFD–D-Lip (H460 and A549: 0.25 mg/mL). Images were captured and processed on days 1, 3, 5, and 9 following treatment.

#### 2.2.11. Mechanism of Action

##### Real-time Annexin Apoptosis Assay

The RealTime-Glo^™^ Annexin V Apoptosis and Necrosis Assay (Promega, Madison, WI, USA) is a live-cell (non-lytic) real-time (kinetic) assay that measures the exposure of phosphatidylserine (PS) on the outer leaflet of the cell membrane during the apoptotic process. Measurement of PS exposure is a reliable and well-validated mean of assessing apoptosis [40]. Briefly, A549 cells were plated at a density of 10,000 cells/well in a white opaque tissue culture 96-well plate (Fisher Scientific, Hampton, NH, USA). The next day, cells were treated with 4X concentration of PFD and PFD–D-Lip, followed by addition of 100 μL of 2X assay buffer and incubated at 37 °C/5% CO_2_. Luminescence signal was monitored at regular time points of 0, 3, 6, 12 h using a Tecan microplate reader [41].

##### Caspase-3 Induction assay

Apoptosis, or programmed cell death, plays a critical role in assessing the potency of drug delivery systems and is a major pathway for cellular death. Caspase-3 activity was measured using an EnzChek^™^ Caspase-3 Assay Kit (Molecular Probes, Eugene, OR, USA) as per the manufacturer’s specifications. Briefly, A549 cells were seeded at a density of 1 x 10^6^ cells/TC dish of a 100-mm diameter dish (Thermo Scientific, Rochester, NY, USA) and treated with PFD or PFD–D-Lip (0.25 mg/mL) for 6 h followed by harvesting and washing of cell pellets. Cell lysis was carried out using 1X cell lysis buffer while subjected to a freeze–thaw cycle, followed by centrifugation. Supernatants obtained were transferred to a 96-well plate to which 50 µL of 2X substrate working solution (10 mM Z-DEVD-AMC substrate + 2X reaction buffer) was added and incubated for 20 min. Then fluorescence was measured at excitation/emission 342/441 nm [42].

##### Muse Annexin V and Dead Cell Assay

Apoptotic cell distribution of A549 cells was assayed by using the MUSE Annexin V & Dead Cell Kit (Millipore, Billerica, MA, USA) according to the manufacturer’s instructions. Briefly, A549 cells were seeded (100,000 cells/well) in a 24 well plate and incubated overnight. The next day, the media was replaced with PFD, PFD–D-Lip (0.1 mg/mL), or fresh media. After 24 h, treatments were removed and cells were collected through trypsinization followed by washing with PBS twice. Cell suspension was diluted with growth media to a concentration of 0.5 x 10^6^ cells/mL. Then, 150 μL of Annexin V/dead reagent and 100 μL of a single cell suspension were mixed in a microtube thoroughly by vortexing for 5 s, followed by incubating in the dark for 20 min at room temperature. Cells were then analyzed using the Muse cell analyzer (Luminex, Austin, TX, USA). The apoptotic ratio was determined by identification of four populations: (i) non-apoptotic cells, not undergoing detectable apoptosis, Annexin V (−) and 7-AAD (−); (ii) early apoptotic cells, Annexin V (+) and 7-AAD (−); (iii) late apoptotic cells, Annexin V (+) and 7-AAD (+); and (iv) cells that have died through non-apoptotic pathway, Annexin V (−) and 7-AAD (+).

##### Western Blot Studies

A549 cells were plated 1 x 10^6^ cells per petri dish and were treated with PFD and PFD–D-Lip (0.25 or 0.5 mg/mL; 24–72 h) at 37 °C/5% CO2. After treatment, the cells were collected and lysed with 1% Triton^®^ X-100 and 1% Halt™ Protease and Phosphatase Inhibitor Cocktail in PBS and bath sonicated for 1 h at 4 °C. Samples were centrifuged for 15 min at 4 °C at 15,000 rpm and lysates were collected. Cell lysate protein was quantified by the DC^™^ Protein Assay Kit (Bio-Rad, Hercules, CA, USA). The samples were mixed with 2x Laemmli buffer and 2-mercaptoethanol, and denatured at 110 °C for 10 min. For Western blot analysis, 10 µg protein was loaded and separated on 4%–20% Mini-PROTEAN^®^ TGX^™^ Precast Protein Gels and transferred to Trans-Blot^®^ Turbo™ Midi PVDF membranes using a Bio-Rad PowerPac™ Basic Power Supply and Trans-Blot^®^ Turbo^TM^ system. The membranes were blocked with 5% bovine serum albumin in PBS and probed with corresponding antibodies overnight at 4 °C. The following antibodies were used: β-actin (PA1-183, Invitrogen), phospho-AKT1 (44-621G, Invitrogen), and β- catenin (13-8400, Invitrogen). Membranes were then incubated with corresponding secondary HRP-conjugated antibodies ab6789 and ab6721 (Abcam) for 1 h at room temperature and subjected to Western bright chemiluminescence (WBF25, The Gel Company, San Francisco, CA, USA). Protein signals were detected on the membranes and were quantified using the chemiluminescent imaging by the Omega Lum^™^ G Imaging System (The Gel Company, San Francisco, CA, USA).

##### Statistical Analysis

All data presented here are mean ± SD or SEM (*n* = 3 to 6). Cytotoxicity studies represent the average of 3 independent trials (*n* = 6 for each trial). Unpaired student’s *t*-test was used to compare two groups, whereas to compare more than two groups, one-way ANOVA followed by Tukey’s post hoc multiple comparison test was used. *P*-value < 0.05 was considered statistically significant.

## 3. Results

### 3.1. UPLC Method Development for PFD

UPLC chromatogram of PFD analysis is presented in Figure S1. Chromatographic resolution capacity of the column was confirmed through analyzing PFD solutions ranging from concentrations of 2 to 50 ppm.

### 3.2. Formulation and Optimization of Pirfenidone Liposomes

**Figure 1:**
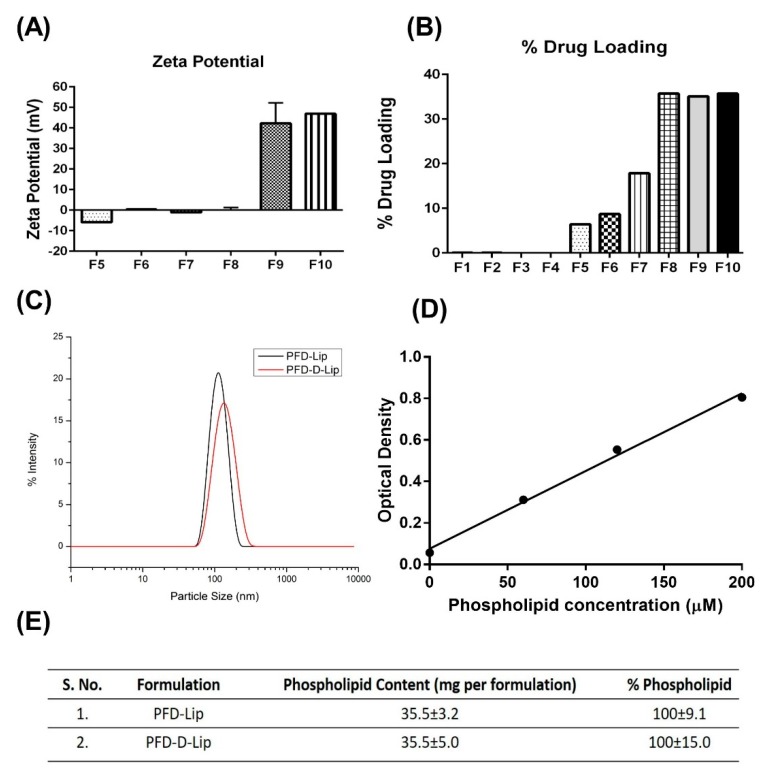
(**A**) Zeta potential of liposomes was determined by a Malvern Zeta Sizer. Zeta potential of F8 and F9 are positive due to the presence of cationic lipid DOTAP. (**B**) % drug loadings were determined for all liposomal formulations. Liposomes formulated using the freeze–thaw technique were found to have the highest amounts of drug loaded. (**C**) Particle size distribution plots of PFD–Lip and PFD–D-Lip. (**D**) Standard curve for phospholipid content performed by using a calorimetric assay (*n* = 4). (**E**) Quantification of phospholipid content in liposomal formulations (*n* = 4).

As shown in Table 1, liposomes formulated by a traditional thin-film hydration method demonstrated optimum particle size (ranging from 120.4 to 179.0 nm), but the entrapment efficiency was minimal (1.1%–2.1%). PFD belongs to biopharmaceutical classification systems (BCS) class II due to its limited aqueous solubility and high permeability. To improve drug loading, a pH gradient approach was also used to incorporate the drug through active loading, which further decreased the drug entrapment (0.25%–0.4%), possibly due to the neutral charge of PFD. Incorporation of PFD into the lipid film, followed by ultra-sonication resulted in a particle size of ~93 nm and 71.7% drug entrapment (F5), which was further improved by using HEPES buffer (pH 7.4) as the film hydration media and a freeze–thaw approach (F6 and F7—almost 100% drug entrapment). As shown in Table 1, increased PFD amount (20 mg) also provided significant drug entrapment (100.58 ± 1.5% for F8 (PFD–Lip); and 98.2 ± 4.7% for F9 (PFD–D-Lip)), while maintaining the particle size at approximately 200 nm, that is essential for efficient cellular uptake. During freeze–thaw cycles, higher encapsulation of actives occurs due to either destabilization or disruption and fusion of lipid film, as described earlier by Costa et al. [26]. It can also be seen that the incorporation of DOTAP, a cationic lipid, imparts a significant positive charge to the formulation (42.2 ± 10 mV for F9 (PFD–D-Lip)), which will provide enhanced cellular uptake of the drug-loaded liposomes (Figure 1A, Table 1). Increasing the amount of DOTAP resulted in increased particle size and polydispersity with no impact on zeta potential or drug loading (F10), and hence this formulation was excluded from further consideration (Figure 1A,B, Table 1).

### 3.3. Characterization of Liposomes

#### 3.3.1. Particle Size, Poly Dispersity Index (PDI), Zeta Potential

As seen in Table 1**,** F8, F9, and F10 were found to exhibit particle sizes ranging from 211.8 ± 12 to 243.2 nm, with a PDI between 0.3 ± 0.07 to 0.4 ± 0.1. F9 and F10 formulations were found to have a positive zeta potential due to the presence of DOTAP, and the different concentrations of DOTAP had no significant effect on the zeta potential values (Figure 1A). Figure 1C represents the uniform particle size distribution with single individual peaks in both PFD–Lip (F8) and PFD–D-Lip (F9) formulations, representing relatively monodispersed liposomal formulations. Standard curve for phospholipid assay and results of phospholipid quantification (PFD–Lip and PFD–D-Lip) are represented in Figure 1D,E, respectively.

#### 3.3.2. Drug Content

As shown in Figure 1B, % drug loading (% of total formulation made up of drug) was found to be significantly higher in formulations prepared by a freeze–thaw approach (F6–F10) and incorporation of cholesterol at a molar ratio equal to DPPC facilitated higher drug encapsulation due to a bilayer-tightening effect established by cholesterol [43], as in case of F5 to F10. The % entrapment efficiency values for F8 and F9 were found to be 100.6 ± 1.5% and 98.2 ± 4.7%, respectively. These results suggest that using a freeze–thaw approach facilitates a higher drug encapsulation in liposomes [26].

#### 3.3.3. In-Vitro Drug Release Studies

**Figure 2:**
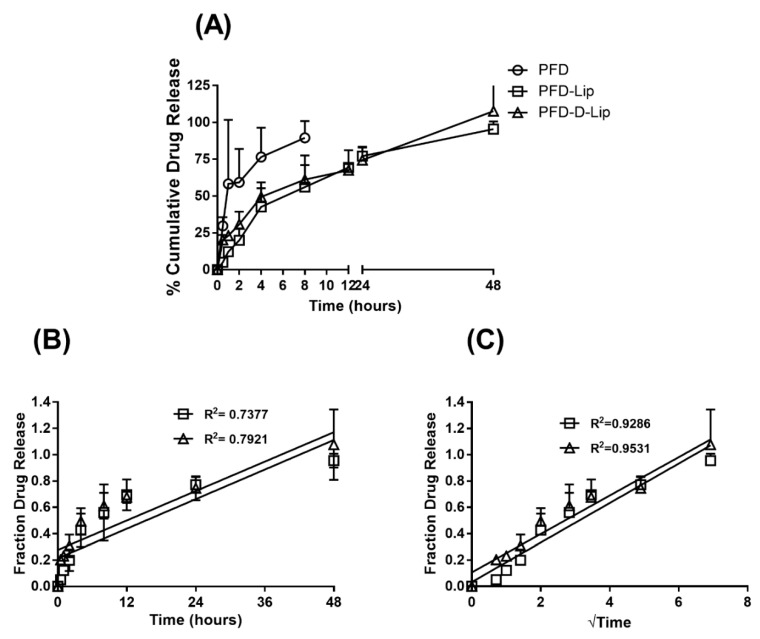
Comparative plots of (**A**) % Cumulative in vitro drug release profile of PFD, PFD–Lip, and PFD–D-Lip, (**B**) zero order release kinetics and (**C**) Higuchi release kinetics from PFD–Lip and PFD–D-Lip in simulated lung fluid (SLF-3 with DPPC liposomes; pH 7.4) at 37 °C. Data represent mean ± SD (*n* = 3). Drug release data was found to best fitted to the Higuchi kinetic model.

The in-vitro release studies were performed to determine the release pattern of pirfenidone from PFD–Lip and PFD–D-Lip in SLF-3 with DPPC liposomes (pH 7.4/37 °C) to mimic the physiological lung fluid environment. Liposomal formulations were found to exhibit a controlled PFD release over 48 h, as shown in Figure 2A. Zero order and Higuchi models or drug release [44] were applied to in-vitro release results and the respective plots are represented in Figure 2B,C. The equations for zero order and Higuchi model release kinetics were found be PFD–Lip: y = 0.01873x + 0.213, PFD–D-Lip: y = 0.01864x + 0.276; PFD–Lip: y = 0.15x + 0.032, PFD–D-Lip: y = 0.146x + 0.106, respectively. Correlation coefficient values were significantly higher (PFD–Lip: R^2^ = 0.9296, PFD–D-Lip: R^2^ = 0.9531) in Higuchi’s model and it was found to be the best fitting model, suggesting that drug transport out of the liposomes was driven mainly by a diffusion-controlled mechanism. The slower release of PFD from both PFD–Lip and PFD–D-Lip is due to sustained release from the inner lamellae of the liposomal formulations.

#### 3.3.4. Morphological Analysis using Transmission Electron Microscopy (TEM)

**Figure 3:**
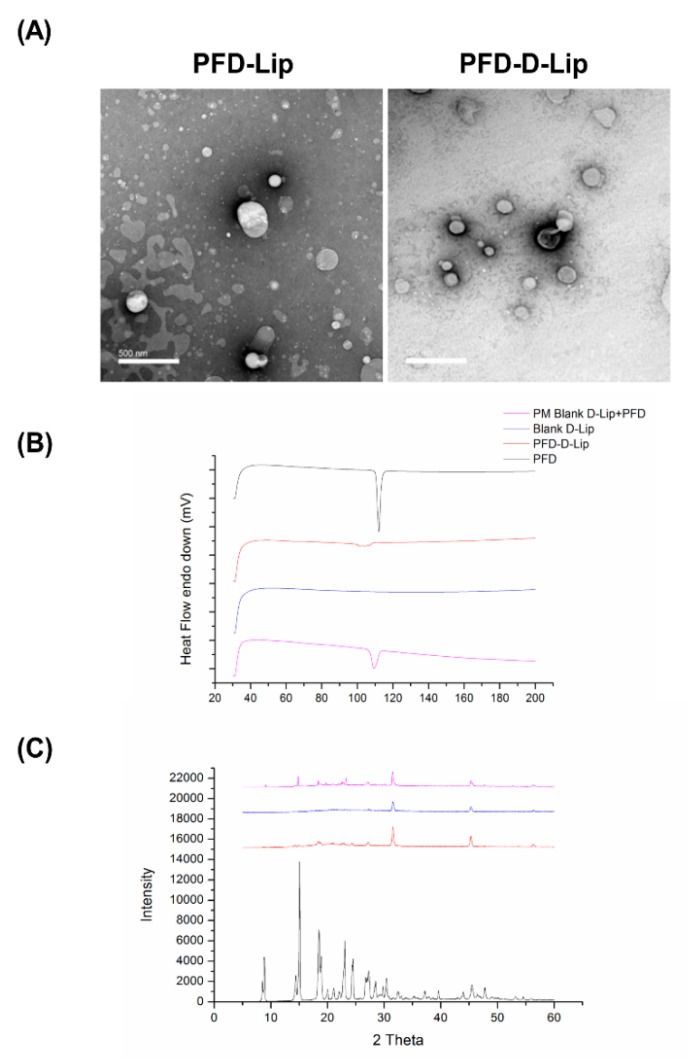
(**A**) Transmission electron microscopy (TEM) images of PFD–Lip and PFD–D-Lip reveal the morphology of different liposomes. Magnification 20 kX. The scale bar represents 500 nm. Vesicles are spherically shaped and uniform size distribution can be observed from PFD–D-Lip. (**B**) Thermograms of pirfenidone, pirfenidone-loaded DOTAP liposomes (PFD–D-Lip), physical mixture of pirfenidone (PFD) and blank liposomes (Blank Lip), and blank liposomes. PFD and physical blend thermograms display a distinct sharp endothermic peak at 112.4 °C in relation to the melting point of the molecule. There is no considerable difference between the thermograms of unloaded and pirfenidone loaded DOTAP liposomes. The absence of the pirfenidone melting peak allows us to suggest that PFD is entrapped inside liposomes, also supported by the XRD data. All experiments were performed in triplicate. (**C**) Indicates the XRD patterns of pirfenidone, pirfenidone-loaded DOTAP liposomes (PFD–D-Lip), physical mixture of pirfenidone (PFD) and blank liposomes (Blank–D-Lip), and Blank–D-Lip. The characteristic sharp crystalline peaks of pirfenidone are evident in the pirfenidone and physical mixture, but not in PFD–D-Lip, indicating the change of crystalline form of PFD to an amorphous state due to encapsulation in liposomes.

TEM images presented in Figure 3A indicate that the liposomes are round to oval shaped, with a smooth surface, representing the presence of unilamellar vesicles. In addition, TEM images also confirmed the vesicle size obtained by DLS measurements. As anticipated based on zeta potential measurements, no aggregation of vesicles was observed during TEM analysis. This demonstrates uniform dispersion of liposomes in the formulation, thus suggesting the presence of a stable liposomal formulation. This information agrees with the data retrieved from the physical characterization results.

#### 3.3.5. Solid State Characterization Studies

Differential Scanning Calorimetry (DSC) Studies: DSC studies were performed to understand the melting and crystallization behavior of PFD, PFD–D-Lip, and a physical mixture of PFD–Lip and Blank Lip. As shown in Figure 3B, a PFD thermogram showed a sharp endothermic peak at 110.40 °C due to its melting transition. In the case of PFD–D-Lip, no sharp peak was observed (Figure 3B). The absence of a sharp peak in PFD–D-Lip indicated the presence of a drug in the molecular dispersion form in the liposomal formulation and suggested consistent results with that of XRD, as discussed below.

Powder X-ray Diffraction (PXRD) Studies: Due to its crystalline nature, PFD showed very large peaks at 2θ values of 8.46, 14.42, 15.04, 18.46, 22.92, 24.36, 26.9, and 32.4 in the XRD spectra. This was in contrast to PFD–D-Lip, which exhibited no peaks, likely due to the amorphous nature of the drug in liposomal formulation (Figure 3C). Results were found to be consistent with earlier studies of PFD microspheres by Soni et al. [45]. The physical mixture of Blank D-Lip and PFD also exhibited peaks inferring to the crystalline nature of the drug.

#### 3.3.6. In-vitro Lung Deposition Studies Using Next Generation Cascade Impactor (NGI)

**Figure 4:**
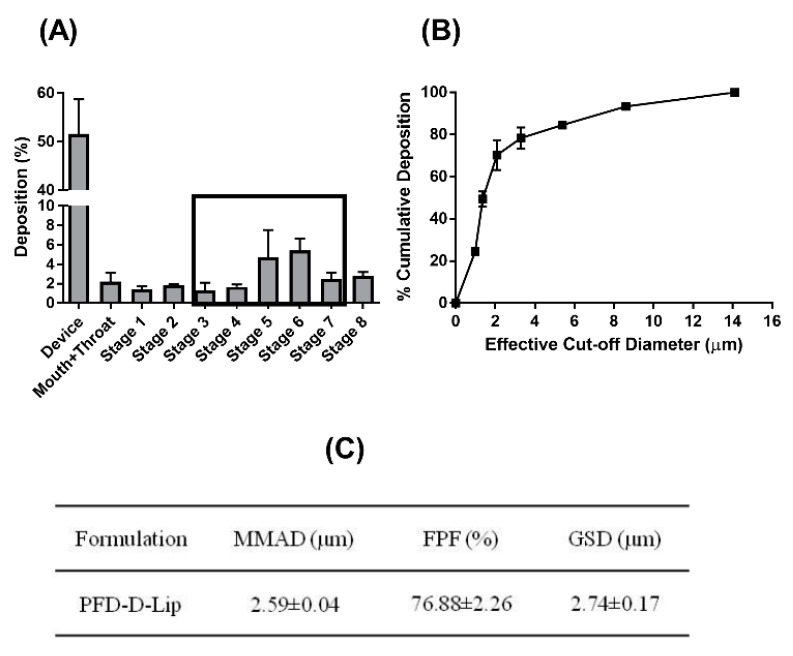
In-vitro deposition profile of PFD–D-Lip. (**A**) Aerosol dispersion performance as % deposited on each stage of the Next Generation Impactor™ (NGI™) for PFD–D-Lip. For Q = 15 L/min for 4 min. The effective cutoff diameters (D_50_) for each impaction stage are as follows: Stage 1 (14.1 µm), Stage 2 (8.61 µm), Stage 3 (5.39 µm), Stage 4 (3.3 µm), Stage 5 (2.08 µm), Stage 6 (1.36 µm), and Stage 7 (0.98 µm). After nebulization of PFD–D-Lip of 2 ml volume, each stage was washed with methanol: ACN (45:55) and the washings analyzed by UPLC to determine the drug deposition (n = 3, average ± SD). (**B**) Cumulative % deposition plot representing cumulative % of particles deposited at each stage. (**C**) Aerosolization properties of PFD–D-Lip.

The deposition of inhaled particles in the respiratory airways depends on several parameters related to the particles (size, charge, density, shape, solubility, and lipophilicity) along with many physiological and anatomical factors of the respiratory system. Deposition behavior and aerosol characteristics can be evaluated by the determination of the aerodynamic behavior of the particles [46]. Once nebulized, aerodynamic properties of particles govern their deposition profile in the airways and alveolar deep lung regions. Using the NGI, crucial parameters to determine respirability of the liposomes were determined. Mass median aerodynamic diameter (MMAD), which describes the median aerodynamic particle size distribution of an aerosol by mass, and GSD, which describes the spread of the aerodynamic particle distribution, were determined. The MMAD of PFD–D-Lip (2.59 ± 0.04 µm) suggests that the majority of the emitted dose will be delivered to the respirable region of the lungs, while the GSD was 2.74 ± 0.17 µm. The FPF, also called the respirable fraction, was 76.88 ± 2.26%, as shown in Figure 4C, which suggests good aerosolization performance. Aerosol dispersion profile representing % cumulative deposition respective to each stage can be seen from Figure 4A,B, respectively [47]. The data obtained suggest that the prepared formulations possess all the characteristics to render them inhalable.

### 3.4. Stability Studies

Stability is a major concern when dealing with the majority of nanocarriers due to emulsion instability, and particle aggregation. As shown in Figure S2, the stability analysis data reveal that PFD–D-Lip formulations were fairly stable at both 4 and 25 °C, with no significant changes in either the particle size or zeta potential (Figure S2C,D). The particle size for PFD–D-Lip was seen to increase significantly at 37 °C, which, being the physiological temperature, would not affect its long-term storage. On the contrary, changes and irregularities were observed in particle size and zeta potential with PFD–Lip at all storage conditions (Figure S2A,B). We could then hypothesize that the positive zeta potential of >20 mV was instrumental in stabilizing PFD–D-Lip, and prevented the particles from coalescing and aggregating [27,48]. No detrimental effect of temperature and storage time was observed on entrapment efficiency either. Hence, the PFD–D-Lip formulation was found to retain its physicochemical properties during their storage at different temperatures over a period of 4 weeks.

### 3.5. Cellular Uptake Studies

**Figure 5:**
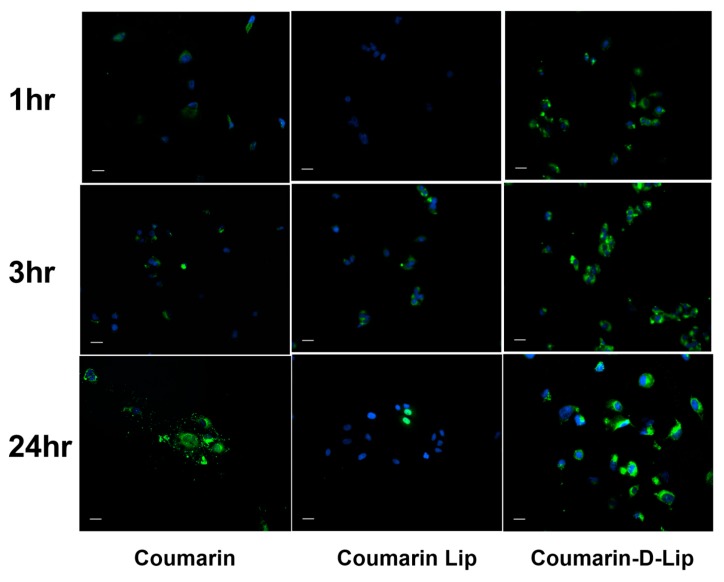
In-vitro cellular uptake of coumarin-6 loaded liposomes, with and without DOTAP in A549 cells at three different time points; 1, 3, and 24 hours. Coumarin-6 plain solution is used as control. Nuclei are stained blue (DAPI) and Coumarin–Lip and Coumarin–D-Lip are green. The highest internalization of Coumarin-loaded DOTAP liposomes was observed at 1, 3, and 24 hr time points compared to plain coumarin and coumarin liposomes. This indicated the importance of cationic charge in DOTAP liposomes in an efficient cellular uptake. Scale bar 100 μm. All experiments were performed in triplicate.

Figure 5 illustrates the intracellular uptake of liposomal formulations in A549 non-small cell lung cancer (NSCLC) cells. For this experiment, PFD was replaced with fluorescent coumarin to allow the visualization of the uptake and accumulation of this formulation. Fluorescent images were taken following 1, 3, or 24 h incubation with Coumarin-loaded liposomes. These images clearly demonstrate that Coumarin–D-Lip provided a significantly higher uptake of coumarin in the A549 cancer cells, as compared to both Coumarin–Lip and plain coumarin within the first 3 h (Figure 5). It was also observed that Coumarin–D-Lip had a higher accumulation around the nucleus, the most desired location for nanoparticle disruption and drug release inside the cells. The presence of a positive charge on Coumarin–D-Lip enables liposomal interaction with cell surfaces (negatively charged), thus further resulting in efficient cellular internalization.

### 3.6. Cytotoxicity Studies

**Figure 6:**
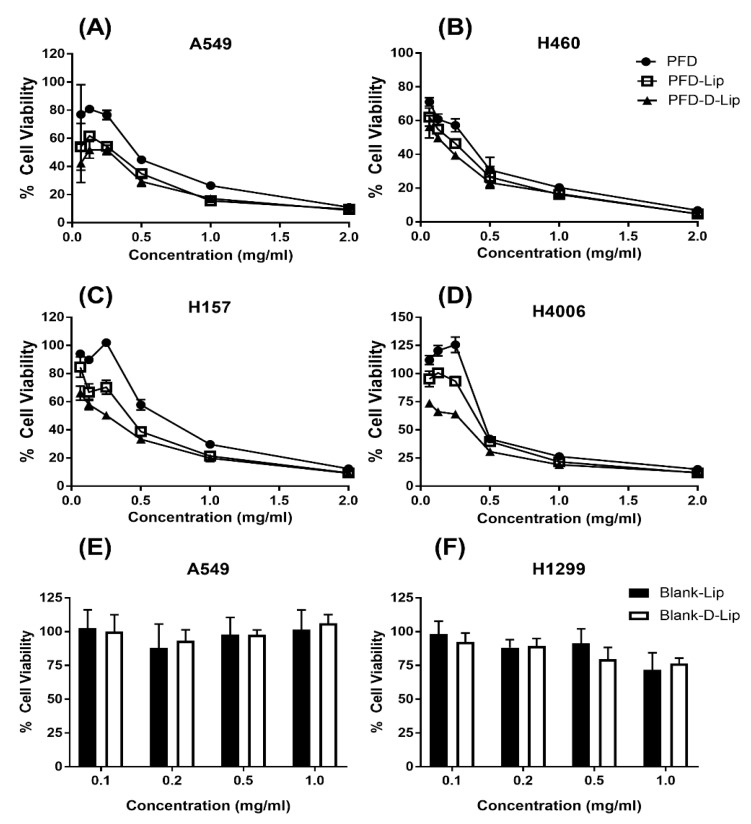
Inhibitory effects on different NSCLC cell lines (**A**) A549, (**B**) H460, (**C**) H157, and (**D**) H4006, after treatments with PFD, PFD–Lip, and PFD–D-Lip. PFD–D-Lip was found to enhance cytotoxicity effects compared to PFD and PFD–Lip. Cells were incubated with different concentrations of PFD, PFD–Lip, and PFD–D-Lip for 72 h; and cell viability was determined using MTT assay. Data represent mean ± SD (n = 3). (**E**,**F**) Safety studies. Cytotoxicity studies on A549 (**E**) and H1299 (**F**) cell lines after treatment with Blank Lip/Blank–D-Lip. Cells were incubated with equivalent amounts of Blank Lip and Blank–D-Lip to 0.1, 0.2, 0.5, and 1mg/mL of PFD–Lip and PFD–D-Lip, respectively, for 72 h; cell viability was determined using MTT assay. Cells without treatment were considered as control (100%). Data represent mean ± SD (*n* = 3).

Cell viability studies were performed using an MTT assay for evaluating the cytotoxic potential of liposomal formulations as compared to pure PFD (Figure 6). From this study, it was found PFD–D-Lip was significantly more cytotoxic than both PFD–Lip and plain PFD. The same trend was observed in all four NSCLC cell lines (A549, H4006, H157, and H460). The IC_50_ values for plain PFD, PFD–Lip, and PFD–D-Lip were found to be 0.43 ± 0.11, 0.37 ± 0.21, and 0.2 ± 0.19 mg/mL in the A549 cell line; 0.45 ± 0.01, 0.42 ± 0.04, and 0.34 ± 0.03 mg/mL in the H4006 cell line; 0.57 ± 0.11, 0.35 ± 0.11, and 0.24 ± 0.08 mg/mL in the H157 cell line; 0.27 ± 0.08, 0.19 ± 0.06, and 0.15 ± 0.09 mg/mL in the H460 cell line, respectively (Table 2).

Smaller particle size and the presence of a positive surface charge of PFD–D-Lip facilitated the efficient internalization of liposomes resulting in more drug accumulation inside the cells and enhanced cell killing. This led to the enhanced cytotoxic potential of the PFD–D-Lip formulation that would also further reduce the dose requirement clinically. In-vitro cytotoxicity studies of Blank–D-Lip were performed on HEK-293 human embryonic kidney cell line. Both Blank–Lip and Blank–D-Lip were found to be non-cytotoxic from % cell viability determinations after incubating A549 and H1299 cell lines for 72 h, which is represented in Figure 6E,F. Moreover, when HEK cell line was treated with 0.1, 0.5, 1, and 2 mg/mL formulation for either 24 or 72 h, in none of the cases was cell viability below 70%, suggesting that formulations were not toxic to HEK cells. These observations were expected since lipids used to prepare liposomes have long been known to produce little or no cytotoxicity. The safety profile of liposomes in the HEK cell line has been presented in Figure S3.

### 3.7. Scratch Assay for Wound Healing and Cell-Cell Interaction

**Figure 7:**
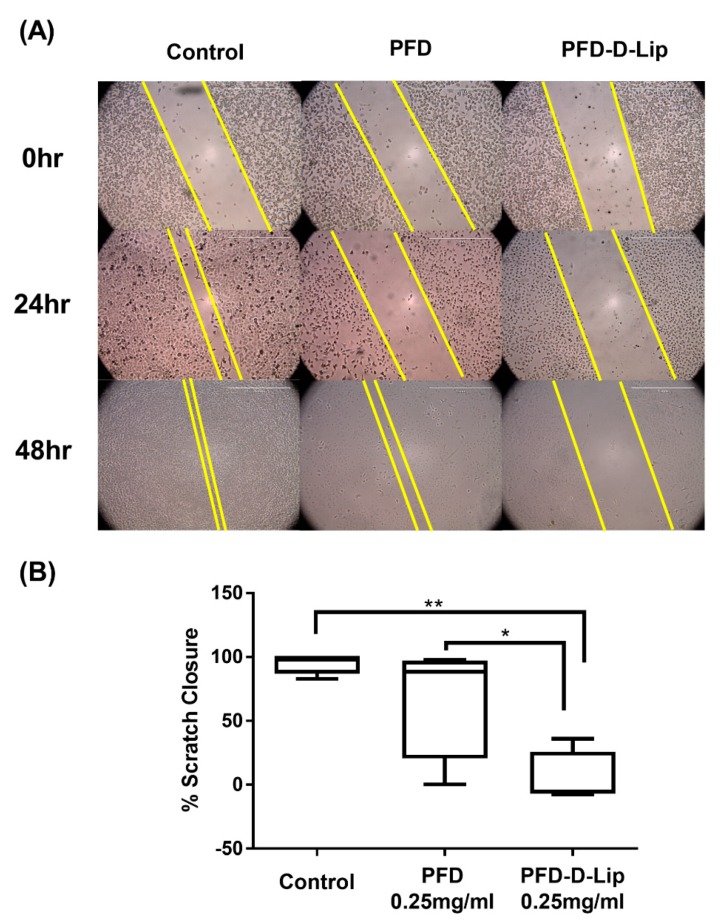
In-vitro scratch wound healing assay with A549 cells treated with PFD and PFD–D-Lip with no treatment as a control. (**A)** shows representative images for indicated treatments taken at 10X magnification using LAXCO microscope. (**B)** Box-whiskers plots show the percent area closure in scratch assay after 48 h. PFD–D-Lip inhibited the migration of A549 cells significantly compared to control and PFD. The uncovered area has been quantified by the ImageJ Software at each time point and represented in the graphs as mean ± SEM of three different experiments. Significance between the groups was analyzed by one-way ANOVA and Tukey’s multiple comparison test. Scale bar 500 μm. * *p* (α < 0.05), ** *p* (α < 0.01).

Scratch assay is a well-established method to assess cell–cell interaction and cellular migration. For these experiments, a scratch was made as described earlier, with cells being treated with either PFD or PFD–D-Lip; and images of the scratched area were taken at 0, 24, and 48 h. Figure 7A represents images after 0, 24, and 48 h of incubation. As shown, there was significant reduction in scratch closure after treatment with both PFD and liposomes. After 48 h, significant cellular migration was observed in both media control and plain PFD groups, with almost no migration in PFD–D-Lip formulation-treated cells (Figure 7A). The results were quantified and the scratch closure was found to be 68.7 ± 24.2% and 4.5 ± 11.1% for PFD and PFD–D-Lip (0.25 mg/mL), respectively, while being normalized to control, as represented in the box-whiskers plot of Figure 7B. Figure 7A,B demonstrates the efficacy of PFD-loaded liposomal formulations in enhancing its anti-migratory effects, which would be instrumental in shrinking tumor volume and reducing the incidences of tumor metastases.

### 3.8. Clonogenic Assay

**Figure 8:**
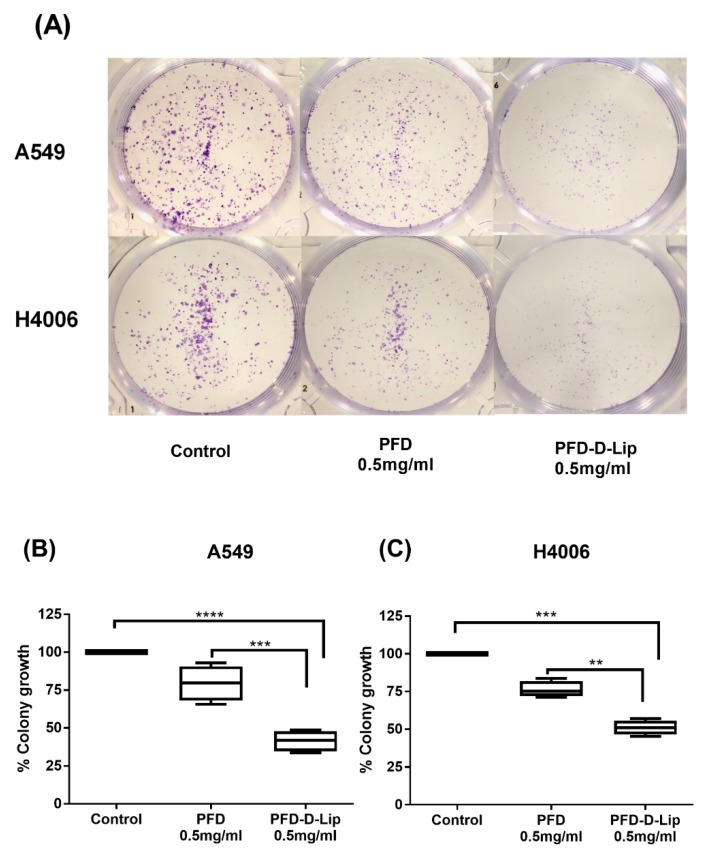
A549 and H4006 cells were treated with control, PFD and PFD–D-Lip at 0.5 mg/mL for 48 hrs. This assay was performed in six-well plates. Treatments were replaced with fresh media and cultured for additional 7 days with media replacement on alternative days. Colonies were then washed with PBS twice, fixed with 4% paraformaldehyde, followed by staining with crystal violet and photographed. (**A**) Representative images showing distinct colonies after staining. Three different experiments were performed. (**B,C**) Quantitative representation of clonogenic assay as % colony growth with PFD or PFD–D-Lip treatment, as compared to control in A549 and H4006 cell lines, respectively. Significance between the groups was analyzed by one-way ANOVA and Dunnet’s multiple comparison test. * *p* (α < 0.05). Data represent mean ± SEM (*n* = 3). ** *p* (α < 0.01), *** *p* (α < 0.001), **** *p* (α < 0.0001).

Clonogenic assay is an in-vitro cell survival assay used to determine the colony formation capability of single cancer cells [49]. PFD and PFD–D-Lip were evaluated for their long-term efficacy against two different NSCLC cell lines (A549 and H4006) in a clonogenic assay. Figure 8A illustrates significant inhibition of colony growth by both plain drug and liposomal formulations, with PFD–D-Lip being significantly better in reducing colony growth in both cell lines (Figure 8). After 48-h drug treatment and 7-day subsequent incubation, the % of colonies surviving after treatment with PFD and PFD–D-Lip were 78.5 ± 4.1% and 41.1 ± 3.0% (A549; *p* < 0.001); and were 78.8 ± 4.7% and 50.7 ± 4.0% (H4006; *p* < 0.01), respectively (Figure 8B,C). This data may be considered representative of PFD–D-Lip’s ability to eliminate the possibility of tumor relapse from single cancer cells left behind following chemotherapy and surgical intervention. This can also be linked to enhanced intracellular (and intratumoral) drug accumulation with liposomal formulation.

### 3.9. In-vitro Angiogenesis Study

**Figure 9:**
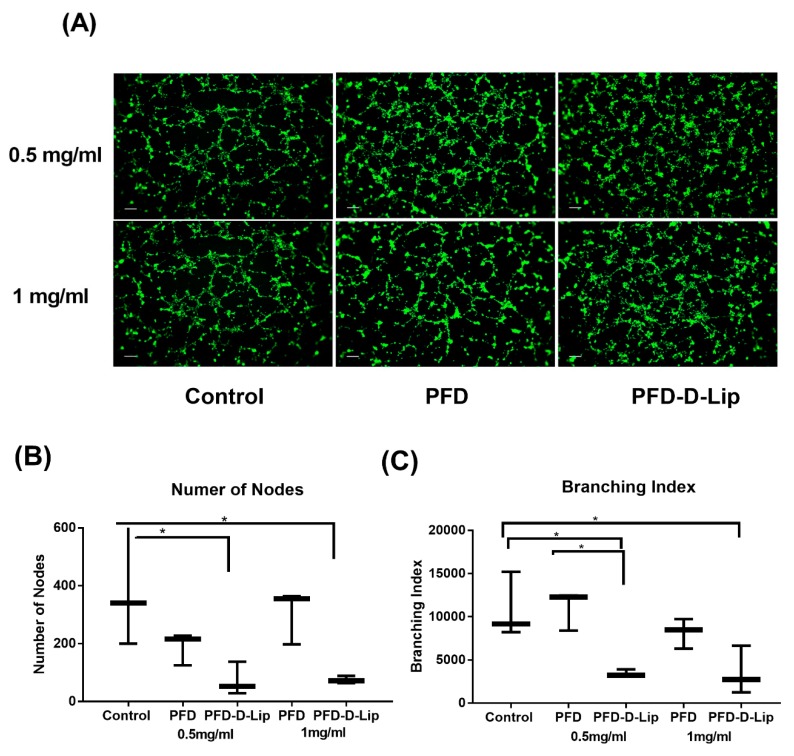
(**A**) Fluorescence microscopy imaging of capillary tube formation by HUVEC cells. Prior to the start of the assay, endothelial cells were treated with 2 µM calcein AM for 30 min to visualize the cells using Evos-FL fluorescence microscopy. Photographs of HUVEC were taken at 4X magnification. (**B,C**) Quantification of tube formation. Number of nodes (**B**) and branching index (**C**) were calculated by quantifying the formed tube network with NIH ImageJ with the Angiogenesis plugin and the results were represented per microscopic field. Significance between groups was analyzed by one-way ANOVA and Tukey’s multiple comparison test. Data are shown as mean ± SEM from three independent experiments. * *p* (α < 0.05). Scale bar 100 μm.

Angiogenesis plays a critical role in the development and progression of many cancers, including NSCLC [50]. Endothelial tube formation or inhibition can easily be determined and quantified by analyzing microscopic images in-vitro. HUVEC vascular endothelial cells were grown on matrigel and were treated with either PFD or PFD–D-Lip at 0.5 and 1 mg/mL for 6 h. As shown in Figure 9A, significant tube formation was observed in both control and PFD treated wells, represented by an organized web of the capillary-like structures (Figure 9A). In comparison, PFD–D-Lip treated cells did not show any organized tube formation and appeared as scattered cells. Tube formation assay images were quantified using NIH ImageJ software with the angiogenesis analyzer plug-in, by measuring the total branch length and number of nodes as represented in Figure 9B,C. PFD–D-Lip showed significant anti-angiogenic potential compared to plain drug at both concentrations. As shown, the number of nodes decreased from 305.3 ± 54.0 (PFD) to 74.7 ± 7.0 (PFD–D-Lip) (0.5 mg/mL; *p* < 0.05), and from 189.3 ± 32.0 to 72.3 ± 33.0 (1 mg/mL, *p* < 0.05). The branching index also reduced significantly from 11,043.3 ± 1320.0 to 3460.7 ± 233.0 (PFD vs. PFD–D-Lip; 0.5 mg/mL; *p* < 0.05); and from 8171.8 ± 1001.0 to 3539.7 ± 1618.0 (1 mg/mL; *p* < 0.05). This reduction provides a rationale for enhanced anti-angiogenic, and thus the tumor inhibiting potential of PFD-loaded liposomes.

### 3.10. 3D Spheroid Cell Culture Study

Three cancer cell lines (A549, H460, and H4006) were evaluated for their ability to form compact spheroids using Nunclon Sphera plates, as previously reported [51]. While A549 and H460 cell lines were able to form spheroid masses from day 1 or day 3 during culture, the H4006 cell line was found to form irregular aggregates or cell clusters.

This study was performed in two separate models: prophylactic and therapeutic. While preventive or prophylactic treatments assist in determining the treatment’s potential for inhibiting tumor growth and may mimic therapy following early diagnosis, a therapeutic model represents traditional cancer therapy focused on reducing an already established tumor mass.

**Figure 10:**
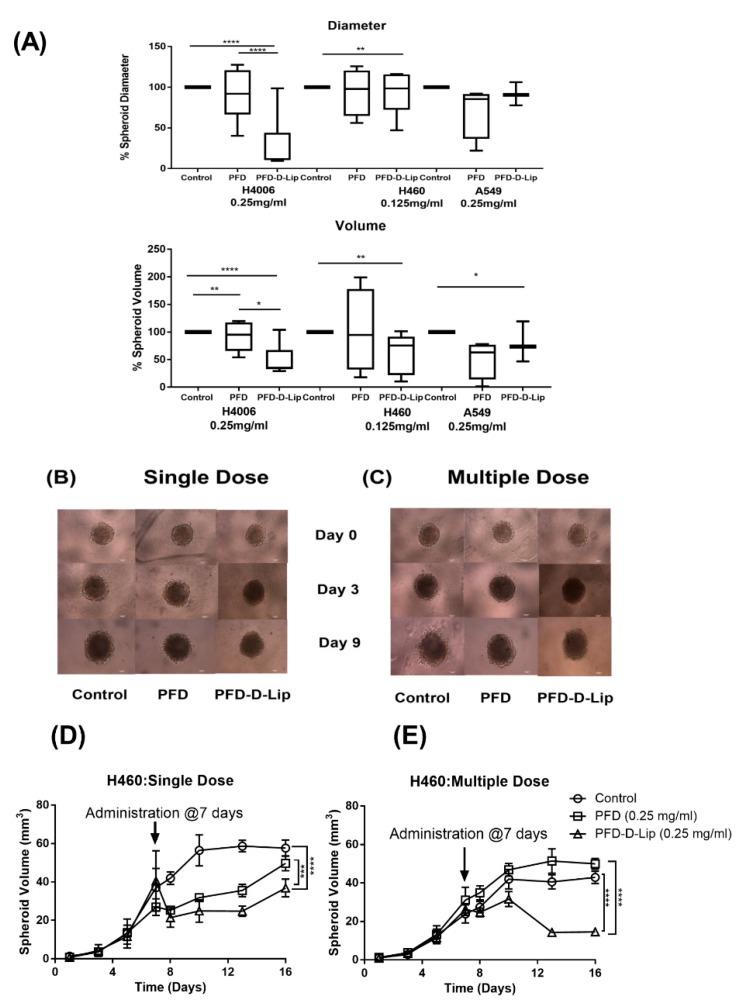
(**A**) Prophylactic model for 3D spheroid study in NSCLC cell lines. A549, H460, and H4006 cells were treated with control, PFD, and PFD–D-Lip (A549: 0.25 mg/mL; H460: 0.125 mg/mL, and H4006: 0.25mg/mL). Spheroid diameter and volume on 15th day of treatment are represented as % reduction from control groups. (**B,C**) Effect of treatment on growth of tumor in A549 3D spheroids after either single (**B**) or multiple dose (**C**). Images represent the spheroids before treatment (day 0) and post treatment (days 3, 9). (**D**,**E**) Spheroids of H460 were treated with PFD–D-Lip showed significantly reduced spheroid size in comparison to control and PFD after 9 days of treatment. Data represent mean ± SD (*n* = 6). Scale bar for the images represents 400 μM. * *p* (α < 0.05), ** *p* (α < 0.01), **** *p* (α < 0.0001).

The prophylactic model involves the plating of cells and overnight incubation followed by next-day treatment. Representative images for the A549 cell line can be seen in Figure S5. The % spheroid diameters and volumes after 10 days of treatment with PFD, PFD–D-Lip (0.25 mg/mL: H4006, A549; 0.125 mg/mL: H460) are shown in Figure 10A. As can be seen, both spheroid diameters and volumes were found to be significantly smaller than control volumes for PFD–D-Lip treated groups. Spheroid diameters (µm) and volumes (mm^3^) after respective treatments were as follows: H4006 (Control: 4141.1 ± 1548.7, 49.3 ± 42.2; PFD: 3495.0 ± 806.3, 25.2 ± 14.3; PFD–D-Lip: 1955.1 ± 848.02, 5.93 ± 7.13); H460 (Control: 4945.23 ± 781.18, 66.8 ± 30.03; PFD: 4500.8 ± 840.3, 51.3 ± 23.6; PFD–D-Lip: 4150.5 ± 931.0, 41.9 ± 22.3).

After normalizing the data to control (set as 100%), spheroid diameters after treating with PFD–D-Lip for H4006 cell line were found to be 47.2 ± 8.4% of control and were also significantly smaller than that of plain PFD treatment groups (*p* < 0.001). Similar results were obtained with H460 and A549 cell lines as well (Figure 10A). Spheroid volume also demonstrated significant reduction in tumor volumes following both plain drug and PFD–D-Lip formulation, with liposomes providing the most efficient reduction in tumor volume (H460: 62.7 ± 13.6% of control, H4006: 12.0 ± 5.9% of control, and A549: 80.5 ± 14.3% of control; Figure 10A). These results reveal that the liposome formulation was able to inhibit spheroid growth in all NSCLC cell lines at clinically relevant doses.

In therapeutic spheroid model, cells were allowed to form a tightly bound cellular mass for 7 days, as would be the case with a late diagnosis of a well-established tumor. After tumor formation, spheroids were treated by either a single dose (one dose in the beginning) or a multiple dose (dosing every third day, mimicking chronic chemotherapy treatments).

As shown in Figure 10B, a tight tumor mass was formed with a diameter and volume of 3860 ± 356.7 µm and 30.9 ± 9.2 mm^3^ (H460) and 1424.1 ± 42.6 µm and 1.5 ± 0.1 mm^3^ (A549), respectively, after 7 days of culture. Then, 9 days after starting the treatment, A549 spheroids from the single dose study had 2.0 ± 0.5 mm^3^ (PFD–D-Lip) and 2.5 ± 0.8 mm^3^ (PFD) volumes where the control spheroid volume was 2.8 ± 1.0 mm^3^ at a 0.25 mg/mL dose. Multiple dosing resulted in reduced spheroid volume to 2.0 ± 0.3 mm^3^ (PFD–D-Lip) compared to the control (3.2 ± 1.2 mm^3^) and PFD (2.7 ± 0.9 mm^3^). A distinct decrease in the spheroid size was observed with both single and multiple dosing. A549 spheroid images after single and multiple doses can be found in Figure 10B,C, respectively. The graphs representing spheroid volume comparison can be found in Figure S4.

In the case of H460 cells, 9 days of a single dose treatment period resulted in spheroids with 36.9 ± 11.4 mm^3^ (PFD–D-Lip) and 49.7 ± 9.5 mm^3^ (PFD) volume, where the control spheroid volume was 57.6 ± 10.3 mm^3^ at 0.25 mg/mL dose. Multiple dosing resulted in reduced spheroid volume to 14.6 ± 3.5 mm^3^ (PFD–D-Lip) compared to control (42.9 ± 7.8 mm^3^) and PFD (42.9 ± 7.0 mm^3^) at 0.125 mg/mL. These results after both single and multiple dosing studies illustrated the efficacy of PFD–D-Lip for tumor volume reduction as compared to no reduction in spheroid volume after plain drug treatment. The graphs representing spheroid volume comparisons can be found in Figure 10D. From the observed efficacy in both spheroid models, it was confirmed that PFD–D-Lip can inhibit tumor growth in earlier as well as later stages.

### 3.11. Mechanism of Action

#### 3.11.1. Real-time Annexin Apoptosis Assay

**Figure 11:**
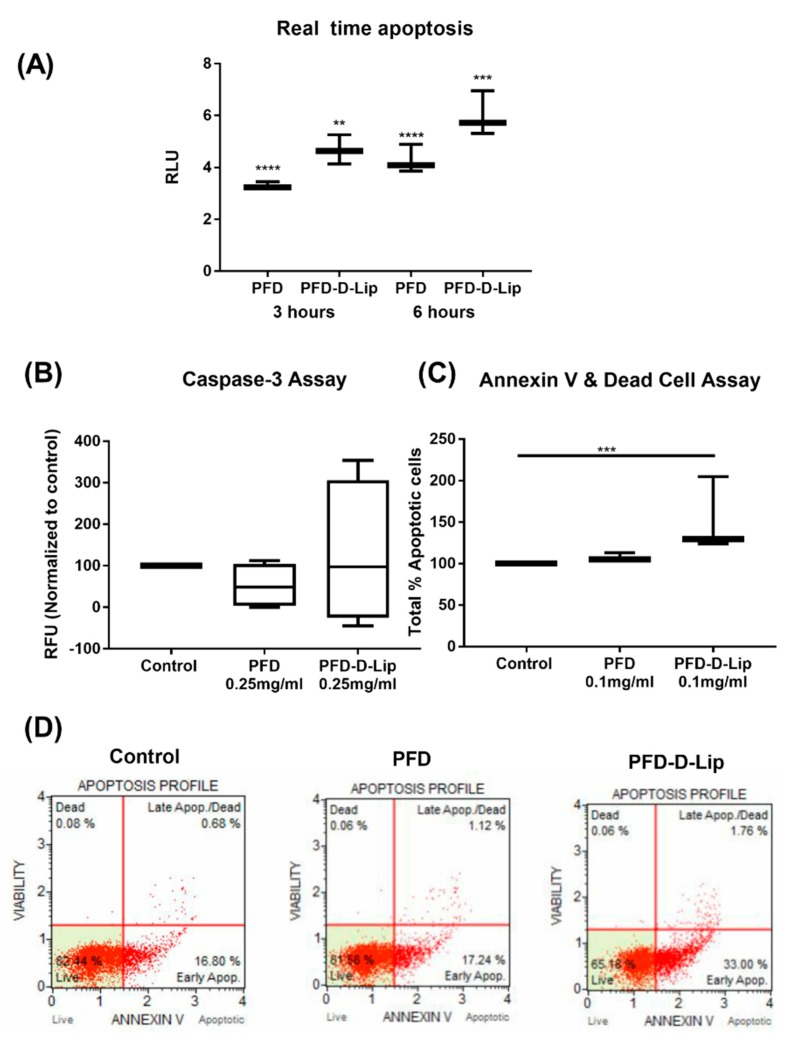
Effects of PFD-loaded liposomes on apoptotic events in A549 cell line. (**A**) Repeated measures of luminescence during exposure to different treatments. A549 cells were exposed to control, PFD, PFD–D-Lip in the presence of the Real Time-Glo™ Annexin V Apoptosis and Necrosis Assay Reagent. Plates were incubated 37 °C/5% CO_2_. RLU were collected at 0, 3, 6, 12 h. Data represent the mean of 3 readings for each treatment ± SEM. Significance between different time points for each treatment groups were analyzed by one-way ANOVA and Tukey’s multiple comparison test. (**B**) Detection of caspase-3 levels in A549 cells using the EnzChek^®^ Caspase-3 Assay. Cells were treated with either 0.25 mg/mL PFD or PFD–D-Lip, and were incubated for 6 hours at 37 °C. Cells were harvested, lysed and assayed. Reactions were carried out at room temperature, and fluorescence was measured in a fluorescence microplate reader using excitation at 360 nm with emission detection at 460 nm after 20 min. Results indicate relative fluorescence units (RFU) normalized to control after each treatment. Data represent the mean of 3 readings for each treatment ± SEM. Significance between the groups was analyzed by one-way ANOVA and Tukey’s multiple comparison test. (**C**) Total % apoptotic profile: graph representing total % of apoptosis when treated with PFD and PFD–D-Lip relative to control. (**D**) Apoptotic impacts of multiple compounds on A549 cell line. Untreated A549 cells were compared with cells treated with PFD and PFD–D-Lip for 24 h using the Muse™ Annexin V & Dead Cell Assay. ** *p* (α < 0.01), *** *p* (α < 0.001), **** *p* (α < 0.0001).

To determine the apoptosis induced in the presence of PFD and PFD–D-Lip, the Real Time-Glo Annexin V apoptosis assay was employed. This assay measures the exposure of phosphatidylserine on the outer leaflet of cell membranes during the apoptotic process through annexin-V binding detected by luminescence, which indicates early apoptosis [52]. It was found that both PFD and PFD–D-Lip (0.5 mg/mL) induced a luminescence signal at the 3-h time point, which increased further until the 6-h time point. Moreover, PFD–D-Lip exhibited significant luminescence signal induction compared to plain PFD, as seen in Figure 11A. This reveals that PFD, after loading into PFD–D-Lip formulation, has exhibited a great potential to induce early apoptosis in A549 cells.

#### 3.11.2. Caspase-3 Induction Assay

To evaluate the induction of active caspase-3 enzyme, cleavage product of a fluorogenic caspase substrate, DEVD–AMC, was measured. The EnzChek^®^ Caspase-3 assay kit allows the detection of apoptosis by measuring the activity of caspase-3 and other DEVD-specific protease activities (e.g., caspase-7). A549 cells treated with liposomes exhibited a higher relative fluorescence intensity normalized to control as compared to treatment with the plain drug, indicating induction of caspase-3 (PFD: 89.36 ± 6.5; PFD–D-Lip: 216.16 ± 7.5 RFU units normalized to control), as seen in Figure 11B. This indicates the capability of PFD–D-Lip in inducing apoptosis and also confirms the anti-cancer activity of PFD–D-Lip, which is enabling apoptosis.

#### 3.11.3. Muse Annexin V Dead Cell Assay

The Muse Cell Analyzer determined the extent of apoptosis in A549 cells incubated with PFD and PFD–D-Lip at 0.1 mg/mL concentration for 24 h. As shown in Figure 11C, total % apoptotic cells were found to be 37.9 ± 1.2% for PFD–D-Lip in comparison with 28.4 ± 5.0% for PFD treatment. The non-treated control exhibited 26.4 ± 4.5% total apoptotic cells. This suggests that PFD–D-Lip resulted in a statistically higher population of total apoptotic cells compared to control (*p* < 0.005). PFD–D-Lip was able to induce early-stage apoptosis even at as low as a 0.1 mg/mL concentration, which led to the highest proportion of total % apoptotic cells. The apoptotic profile of A549 cells after incubating with respective treatments is shown as scatter plots in Figure 11D. Here, the right shift of the scatter plot can be observed in the case of PFD–D-Lip compared to that of the control and PFD.

#### 3.11.4. Western Blot Studies

**Figure 12:**
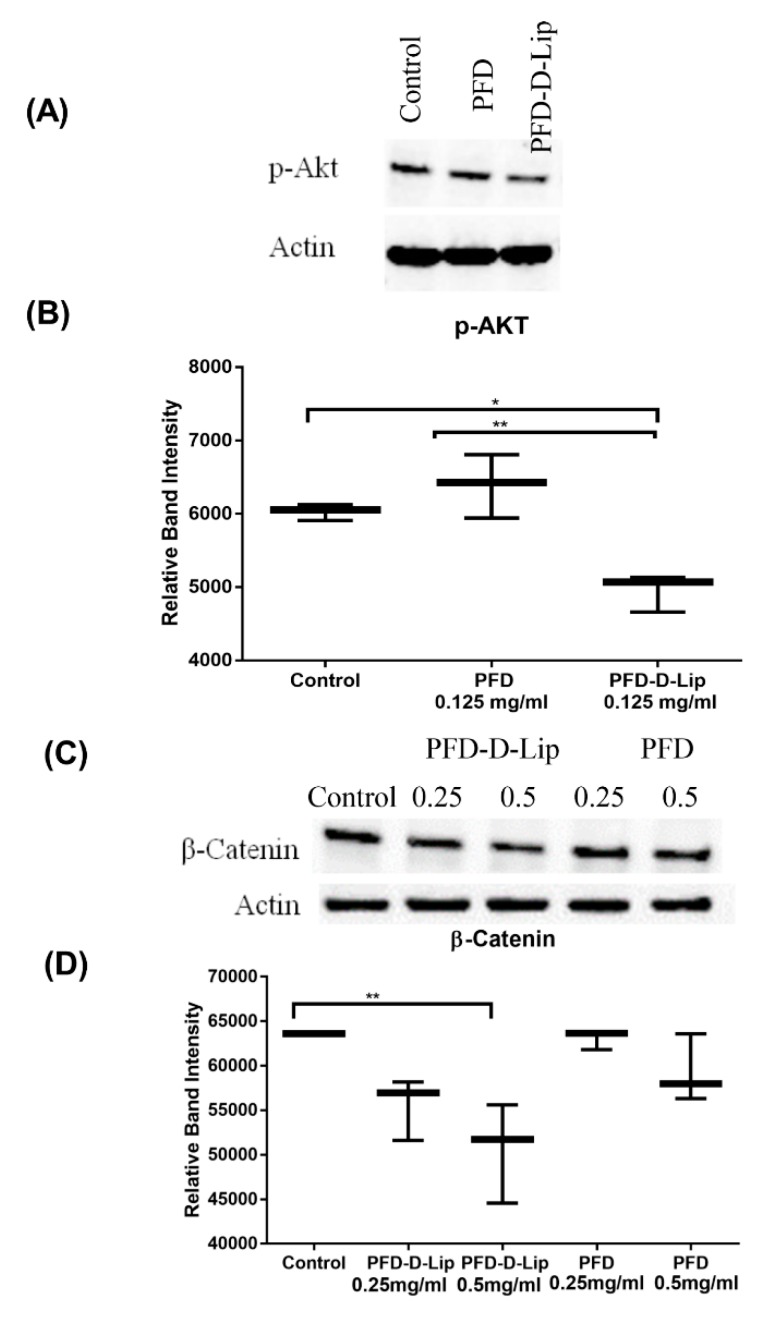
(**A**) Western blot representing inhibitory effect of treatments on expression of p-AKT protein in A549 cells. (**B)** PFD-D-Lip significantly suppresses p-AKT expression in A549 cells. Cell lysates were prepared following 24-hour treatment of A549 cells with control, PFD and PFD-D-Lip (0.125 mg/mL) and were subjected to western blot analyses using antibodies against p-AKT and actin as a loading control, as indicated. (**C**) Western blot representing inhibitory effect of treatments on expression of β-catenin protein in A549 cells. (**D**) PFD-D-Lip suppresses β-catenin expression in A549 cells. Cell lysates were prepared following 72-hour treatment of A549 cells with control, PFD and PFD-D-Lip (0.25 and 0.5 mg/mL) and were subjected to western blot analyses using antibodies against β-catenin and actin as a loading control, as indicated. The western blot densities were determined and plotted as relative band intensity against treatments. Western blot bands represent representative image of n = 3. Data represent mean ± SD (*n* = 3). * *p* (α < 0.05), ** *p* (α < 0.01).

β-catenin and p-AKT play a major role in cancer progression, as previously reported [53,54]. β-catenin and p-AKT are involved in a signaling cascade and their inhibition is an indication of apoptotic induction [41]. The expression levels of β-catenin and p-AKT proteins were determined using Western blot analysis and the representative blots can be found in Figure 12A–D. As shown, PFD–D-Lip significantly downregulated the levels of p-AKT and β-catenin after 24- and 72-hour treatments, respectively, indicating induction of apoptosis. This may be the most important pathway through which PFD and PFD–D-Lip illicit their enhanced tumor-obliterating effects.

## 4. Discussion

Liposomal drug delivery systems are one of the most promising non-toxic carriers of both lipophilic and hydrophobic drugs [28,55]. These systems enhance the therapeutic index of drugs by increasing drug uptake, resulting in increased concentration in tumor cells [56]. The thin-film hydration technique has been widely used for liposomal preparation. Due to the low solubility of pirfenidone, it was difficult to encapsulate it in the aqueous core of liposomes by thin-film hydration. To improve the drug loading, a pH gradient approach was used; however, this did not impact the drug loading, possibly due to the absence of surface charge on pirfenidone. Furthermore, to improve drug entrapment, liposomes obtained after ultrasonic probe sonication were subjected to a freeze–thaw technique, which significantly improved drug entrapment, in line with earlier published reports [28,57]. A particle size of 211.8 ± 12.0 nm with a ≈100% drug entrapment was achieved in the case of PFD–D-Lip (Formulation F9, Table 1). Results of lipid content quantification from PFD–Lip and PFD–D-Lip have revealed great recovery of phospholipid even after several steps of the formulation. Hence, the drug loading efficiencies were not over-estimated. The presence of a positive charge on the surface of PFD–D-Lip refers to the stability of liposomes through inhibiting aggregation [58]. TEM images represented in Figure 3A revealed that the liposomes were of spherical shape with a smooth surface and little to no aggregation, indicating that the vesicles were uniformly dispersed. XRD and DSC results were found to be consistent with earlier studies where a polymeric network of microspheres made of pirfenidone was formulated [45].

The presence of a positive charge on the liposomal surface incorporates a greater capacity to be internalized due to their potential interaction with the negatively charged cell membrane [59]. In a recent study by Kang et al., it was reported that cationic liposomes exhibited higher uptake due to their potential electrostatic interaction with cells [60]. The results of our cellular uptake studies were in agreement with these findings. The aggregation of liposomes in the formulation over a period of time is a common issue found in regards to their stability [27]. Hence, studies were conducted to determine the stability of formulated liposomal carriers. Results revealed that there was no significant difference in particle size, zeta potential, and drug entrapment over a period of 4 weeks at a typical storage temperature of either 4 or 25 ^°^C, thus underlining the robustness and utility of the formulation development process. From the results of in-vitro release studies in simulated lung fluid and their kinetic modeling, it can be understood that the release of PFD from the liposomes followed the Higuchi model, inferring to a diffusion-controlled drug release mechanism [44,61].

The pulmonary route of administration is well known for its ability to deliver therapeutics locally to the site of action but is challenged by major obstacles like poor control over deposition rates and site of inhaled molecules for efficacious delivery at the same time. Liposomes exhibiting optimal in-vitro aerosol lung deposition can be administered via inhalation, facilitating local delivery to the deep lungs, thus resulting in reduced exposure to other organs and reduced adverse events. Nebulizers can deliver the formulations as small droplets, which will be deposited in the lung airways based on their MMAD [62]. In the current study, MMAD and %FPF values confirmed the respirability of formulated liposomes and efficient lung deposition. Developed PFD–D-Lip are capable of providing a promising strategy to overcome the physiological barriers involved in the respiratory tract and ensuring their capability to reach the respirable regions of the lung [62].

In a recent study, Mediavilla-Valera et al. reported pirfenidone to have a direct effect on NSCLC tumor cell proliferation in synergy with cisplatin. However, they reported a modest reduction in A549 cell viability following 72 h PFD (0.5 mg/mL) treatment, along with in-vivo efficacy of the synergistic PFD/cisplatin combination [16]. This study suggested the anti-NSCLC potential of PFD at a higher dose, which could be improved by utilizing cell permeating drug delivery systems. In another study, Kozono et al. showed that pirfenidone inhibited proliferation and cell invasion in pancreatic stellate cells in-vitro and suppressed desmoplasia to exert anti-tumor effects against pancreatic cancer. In addition, they also reported PFD’s efficacy to effectively suppress orthotopic tumor growth in combination with gemcitabine [63]. Moreover, PFD was reported to exert synergistic effects on NSCLC tumor growth in combination with carboplatin in a study published by Fujiwara et al. [17]. In the present study, liposomal formulations of PFD were found to be effective and exhibited significantly enhanced cytotoxicity in various NSCLC cell lines alone at minimal concentrations compared to previous studies.

Cellular proliferation and migration are vital for the initiation and development of many cancers. In specific studies, pirfenidone has been shown to inhibit the proliferation of tumor cell types, such as human leiomyoma cells, and suppressed invasiveness and migration of pancreatic cancer cells, human lens epithelial cells, and retinal pigment epithelial cells while multi-target potential of PFD was reported to result in promising anti-migratory effect [53,63,64,65]. In the present study, results from scratch cell migration, and colony formation assays revealed that PFD-loaded liposomes were more effective in reducing cellular interaction, migration, and single-cell tumor development, as compared to plain drug, which makes it suitable to be considered as an alternative for repurposing PFD for lung cancer. This study also reveals the importance of a developed liposomal system in preventing tumor recurrence due to their long-term efficacy in inhibiting colony formation. The safety profile of Blank–Lip and Blank–D-Lip has been studied through determining cytotoxicity effect on NSCLC cell lines (A549, H1299) and the human embryonic kidney (HEK-293) cell line, indicating the non-toxic nature of the blank formulations.

The tube formation assay is a fast, reproducible, and sensitive method for in-vitro measurement of angiogenesis, a vital component of tumor development [66]. In a recent study conducted by Liu et al., PFD was reported to demonstrate anti-angiogenic activity in-vitro through VEGF-A/VEGFR-2 and its downstream AKT signaling pathway, so as to retard the wound healing (pro-proliferation) process [67]. Liposomal PFD was not only capable of providing anti-angiogenic effects, it completely disrupted HUVEC cellular arrangements, resulting in non-existent vascular tube formations and scattered cellular geometry. This provides another important reason to further evaluate PFD-loaded liposomes in treating cancer patients so as to reduce tumor mass formation by cutting off the blood and nutrient supplies to the tumor mass.

The development of 3D in-vitro models is of utmost necessity today to mimic in-vivo solid tumor conditions, which could be achieved by using specific low attachment spheroid plates as reported by Vaidya et al. [51]. Mesothelioma cell migration/invasion through a collagen matrix, as well as invasive growth into 3D collagen, was found to be decreased by pirfenidone treatment in a recent study [68]. Stealth PEGylated doxorubicin liposomes (Doxil^TM^) have been approved for ovarian cancer and are also assessed as an adjuvant therapy for small cell lung cancer [69]. In another study carried out by Zhai et al., transferrin-targeted docetaxel liposomes were reported to exert enhanced anti-cancer efficacy [70]. Chen et al. have developed liposomes of β-Elemene and reported a significant antitumor activity [71]. From these results, it is understood that liposomes can deliver the drug not only to cells on the tumor surface, but they can also penetrate deep into the spheroids’ core to efficiently provide tumor reduction. In a recent study reported by Zou et al., pirfenidone was found to promote apoptosis in hepatocellular carcinoma cells, which was confirmed by Western blot analysis [53]. In another study by Komiya et al., it was reported that PFD could be repurposed for human non-alcoholic steatohepatitis [72].

Activation of the PI3K-AKT-mTOR pathway has been reported to be a central event in carcinogenesis [73]. Phosphorylated AKT (p-AKT) leads to a signaling cascade causing inactivation of components in apoptosis such as caspases [54]. Moreover, AKT has been implicated in regulating angiogenesis, another critical process in tumor development. β-catenin is another protein that plays a crucial role in Wnt/β-Catenin resulting in cellular proliferation [74]. Sun et al. demonstrated that PFD was able to inhibit the proliferation of human intestinal fibroblasts and induce apoptosis through inhibition of the Smad and PI3K/AKT pathways [75]. Results from the present study indicate that PFD–D-Lip can inhibit both p-AKT (Figure 12A) and β -catenin (Figure 12C) effectively which can be correlated with the increased caspase levels observed from apoptosis assay and inhibited angiogenesis.

Repurposing of anti-fibrotic drugs for cancer treatment could be a promising approach to interrupt the tumor growth cascade through multiple modulatory pathways. While most reports on PFD have studied fibroblasts, with few reports highlighting its effect on cancer progression, it is well known that PFD has the potential to be developed as an anti-cancer therapy. However, the dose of PFD remains very high, resulting in adverse events. In the current study, the efficacy of pirfenidone liposomes against NSCLC has been investigated and confirmed through in-vitro cell culture models as well as in an ex-vivo 3D-spheroid model, illustrating increased cell death and tumor regression in NSCLC. These studies provide several pieces of evidence that, together with published results, indicate that pirfenidone as a liposomal drug delivery system is efficacious in treating NSCLC.

## 5. Conclusion

This report establishes that liposomal formulations are of optimal particle size, drug entrapment efficiency, and efficient respirability. It can be concluded that PFD-loaded liposomes while being inhalable, present a potential treatment strategy for NSCLC and also a superlative example of successful collaboration between novel drug discovery and delivery methodologies in providing practical solutions for cancer epidemic and associated challenges. While the results are promising for effective uptake of liposomes by cells, and demonstrate significant cytotoxic effects, more mechanistic and preclinical/clinical studies are required to encompass the full spectrum of this approach’s feasibility. Further studies along these lines will emphasize the drug repurposing of pirfenidone to treat NSCLC.

## Figures and Tables

**Table 1:** Optimization and Characterization of Liposomal Formulations.

Code	Molar ratio and Total Lipid (mM)	Technique	Drug Quantity	Hydrating Medium	Particle Size (nm)	PDI	Zeta Potential (mV)	% Entrapment
F1	7:3 (20 mM)	Passive; Extrusion	5 mg	2.5 mg drug/mL water	179.0	0.156	-	2.135
F2	5:5 (20 mM)	Passive; Extrusion	5 mg	2.5 mg drug/mL water	120.4	0.120	-	1.138
F3	7:3 (20 mM)	Active pH 3.0; Extrusion	5 mg	Water	582.4	0.232	-	0.251
F4	5:5 (20 mM)	Active pH 3.0; Extrusion	5 mg	Water	138.1	0.038	-	0.404
F5	5:5 (10 mM)	Passive; Ultra Probe sonication	5 mg	Water	92.74	0.202	-5.9	71.7
F6	5:5 (10 mM)	Passive; Ultra Probe sonication and Freeze-thawed	5 mg	HEPES buffer	120.9	0.350	0.369	97.7
F7	5:5 (10 mM)	Passive; Ultra Probe sonication and Freeze-thawed	10 mg	HEPES buffer	187.7	0.383	-1.13	106.7
F8 (PFD–Lip)	5:5 (10 mM) Regular	Passive; Probe sonication and Freeze-thawed	20 mg	HEPES buffer	214.08 ± 7.3	0.4 ± 0.1	0.02 ± 1.3	100.58 ± 1.5
F9 (PFD–D-Lip)	5:5 (10 mM) DOTAP (5%)	Passive; Probe sonication and Freeze-thawed	20 mg	HEPES buffer	211.8 ± 12	0.3 ± 0.07	42.2 ± 10	98.2 ± 4.7
F10	5:5 (10 mM) DOTAP (10%)	Passive; Probe sonication and Freeze-thawed	20 mg	HEPES buffer	243.2	0.323	46.9	104.7

**Table 2:** IC_50_ of Pirfenidone, PFD–Lip and PFD–D-Lip in four NSCLC cell lines.

Cell Line	Pirfenidone	PFD–Lip	PFD–D-Lip
A549	0.43 ± 0.11	0.37 ± 0.21	0.2 ± 0.19
H4006	0.45 ± 0.01	0.42 ± 0.04	0.34 ± 0.03 ^a, b^
H157	0.57 ± 0.11	0.35 ± 0.11	0.24 ± 0.08 ^c^
H460	0.27 ± 0.08	0.19 ± 0.06	0.15 ± 0.1

Significance: a **, b *, c *.

## Supplementary Materials

The following are available online at https://www.mdpi.com/1999-4923/12/3/206/s1, Figure S1: Representative chromatogram of Pirfenidone obtained using Acquity UPLC (Waters, USA). Coulmn: Xselect^®^ CSH C18 (3.0 × 50 mm; 2.5 μm particles); mobile phase: aqueous phase of HPLC grade water of pH 4.5 adjusted with orthophosphoric acid, and organic phase of methanol and ACN (45:55) at 60:40; flow rate: 0.4 mL/min at 210 nm wavelength. Retention time was found to be 1.3 min with total run time of 4 min, Figure S2: Influence of storage temperature and length of storage on particle size and zeta potential of both PFD-Lip (A & B); and PFD-D-Lip (C & D). Formulations were stored at 4°, 25° and 37 °C over a period of 4 weeks. Data represent mean ± SD (*n* = 3), Figure S3: Safety studies: Cytotoxicity studies on HEK cell line after treatment with Blank-D-Lip. Cells were incubated with equivalent amounts of blank DOTAP liposomes to 2mg/ml of PFD-D-Lip for 24 and 72 hours; cell viability was determined using MTT assay. Cells without treatment were considered as control (100%). Data represent mean ± SD (*n* = 6), Figure S4: Therapeutic spheroid study: Spheroid size comparison for single versus multiple dose treatments on day of 0, 3 and 9 of post-treatment. Spheroids of A549 treated with PFD-D-Lip showed significantly reduced spheroid diameter and volume in comparison to control and PFD treated groups after 15 days of treatment. Data represent mean ± SD (*n* = 6), Figure S5: Prophylactic spheroid study: Representing spheroid images on day 3, 5, 7 10 and 15 after treating with control, PFD-Lip and PFD-D-Lip (0.25 mg/ml). A significant reduction was observed in the diameter and volumes of spheroids in presence of PFD-D-Lip as compared to PFD and control. Data represent mean ± SD (*n* = 6).
