# Supplementary material for: Systematic Development and Optimization of Inhalable Pirfenidone Liposomes for Non-Small Cell Lung Cancer Treatment
Source: Pharmaceutics. 2020 Feb 28;12(3):206. doi: 10.3390/pharmaceutics12030206 (PMC7150896; doi:10.3390/pharmaceutics12030206)
Supplement: Supplementary file 1 [file pharmaceutics-12-00206-s001.pdf]

Supplementary Materials: Systematic Development and Optimization of Inhalable Pirfenidone Liposomes for Non-Small Cell Lung Cancer Treatment

Vineela Parvathaneni, Nishant S. Kulkarni, Snehal K. Shukla, Pamela T. Farrales, Nitesh K. Kunda, Aaron Muth and Vivek Gupta *

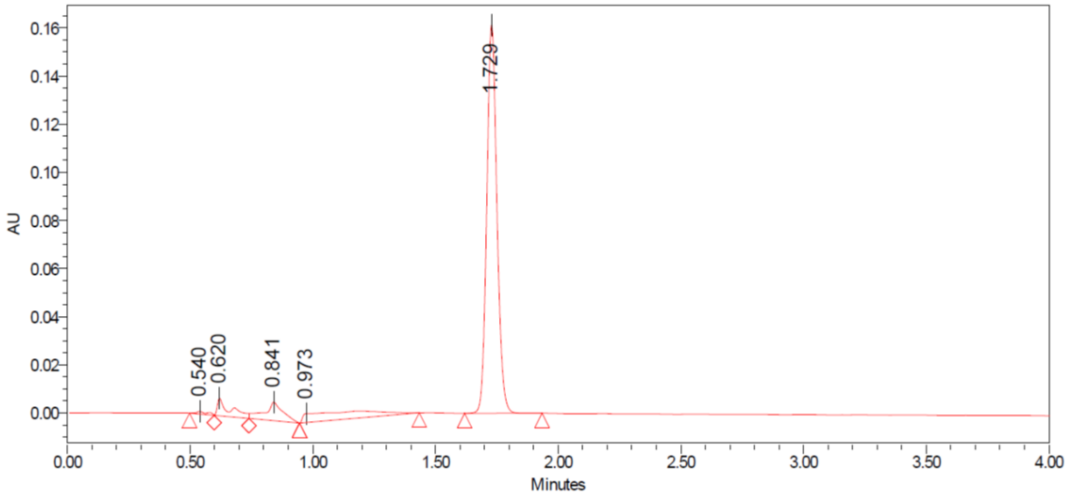

Figure S1. Representative chromatogram of Pirfenidone obtained using Acquity UPLC (Waters, USA). Coulmn: Xselect® CSH C18 (3.0 × 50 mm; 2.5 µm particles); mobile phase: aqueous phase of HPLC grade water of pH 4.5 adjusted with orthophosphoric acid, and organic phase of methanol and ACN (45:55) at 60:40; flow rate: 0.4 mL/min at 210 nm wavelength. Retention time was found to be 1.3 min with total run time of 4 min.

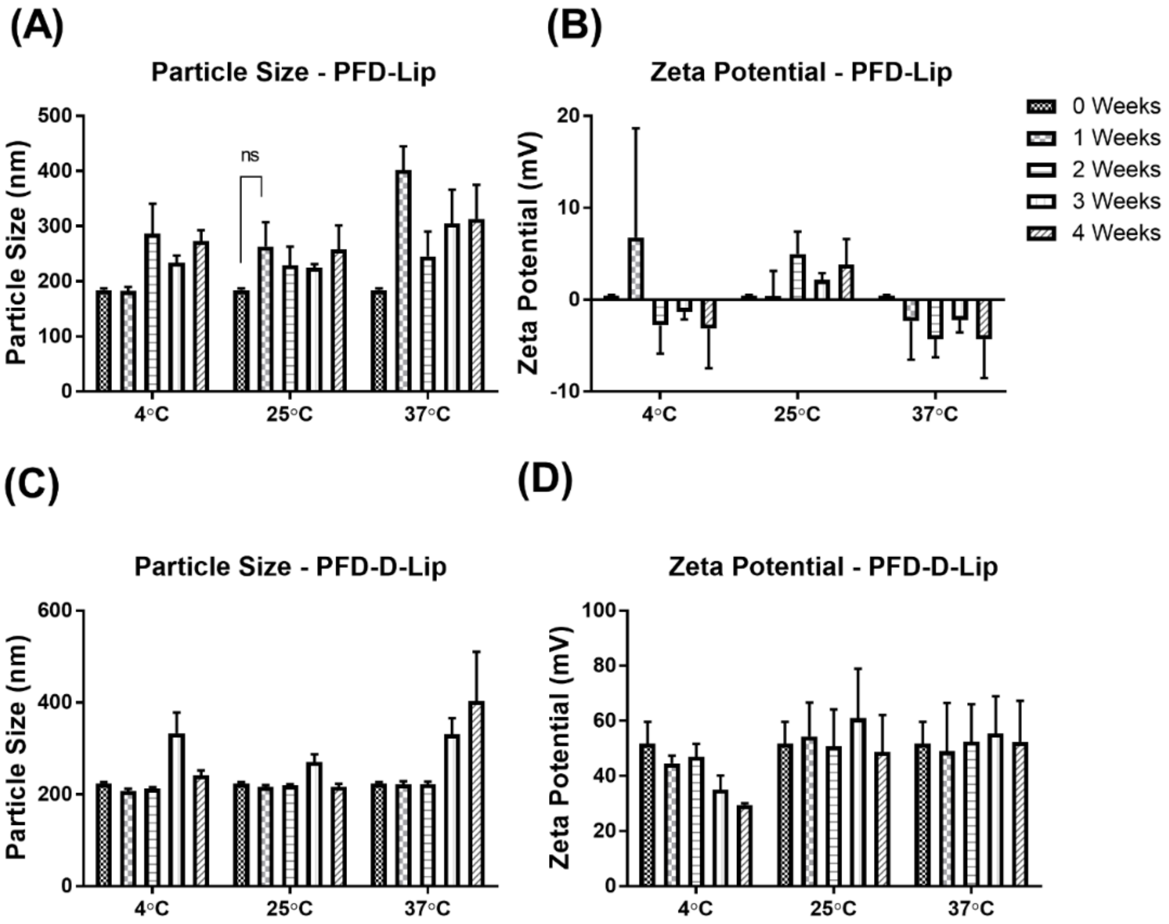

Figure S2. Influence of storage temperature and length of storage on particle size and zeta potential of both PFD-Lip (A & B); and PFD-D-Lip (C & D). Formulations were stored at 4, 25 and 37 °C over a period of 4 weeks. Data represent mean ± SD (n = 3).

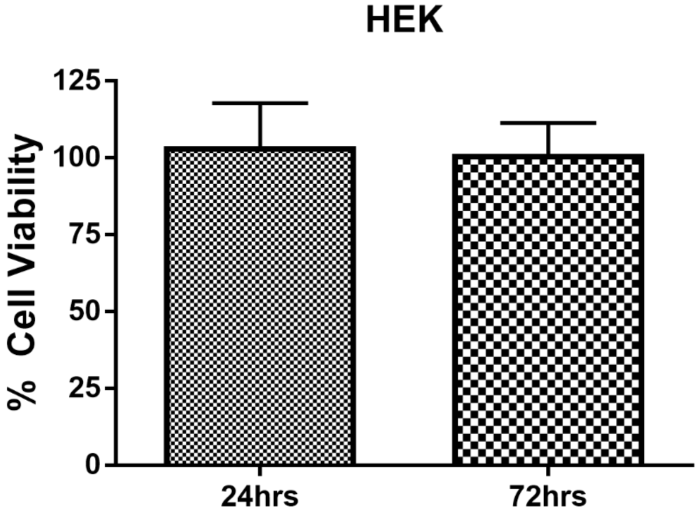

Figure S3. Safety studies: Cytotoxicity studies on HEK cell line after treatment with Blank-D-Lip. Cells were incubated with equivalent amounts of blank DOTAP liposomes to 2mg/ml of PFD-D-Lip for 24 and 72 hours; cell viability was determined using MTT assay. Cells without treatment were considered as control (100%). Data represent mean ± SD (n = 6).

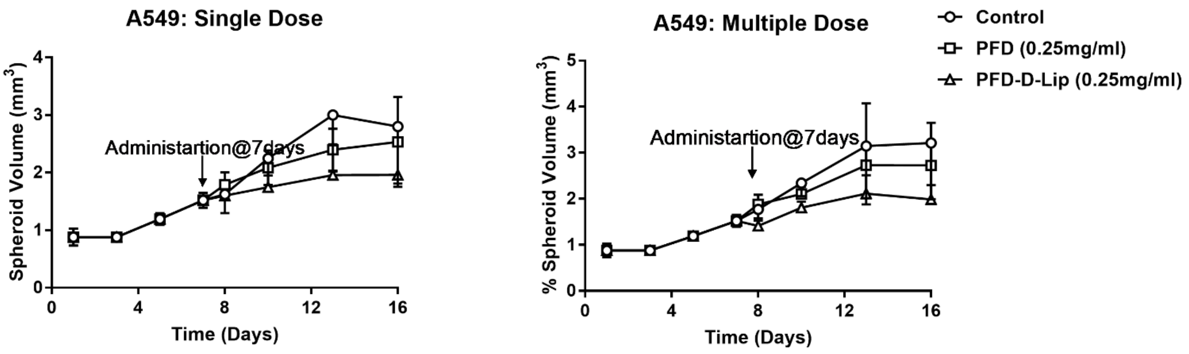

Figure S4. Therapeutic spheroid study: Spheroid size comparison for single versus multiple dose treatments on day of 0, 3 and 9 of post-treatment. Spheroids of A549 treated with PFD-D-Lip showed significantly reduced spheroid diameter and volume in comparison to control and PFD treated groups after 15 days of treatment. Data represent mean ± SD (*n* = 6).

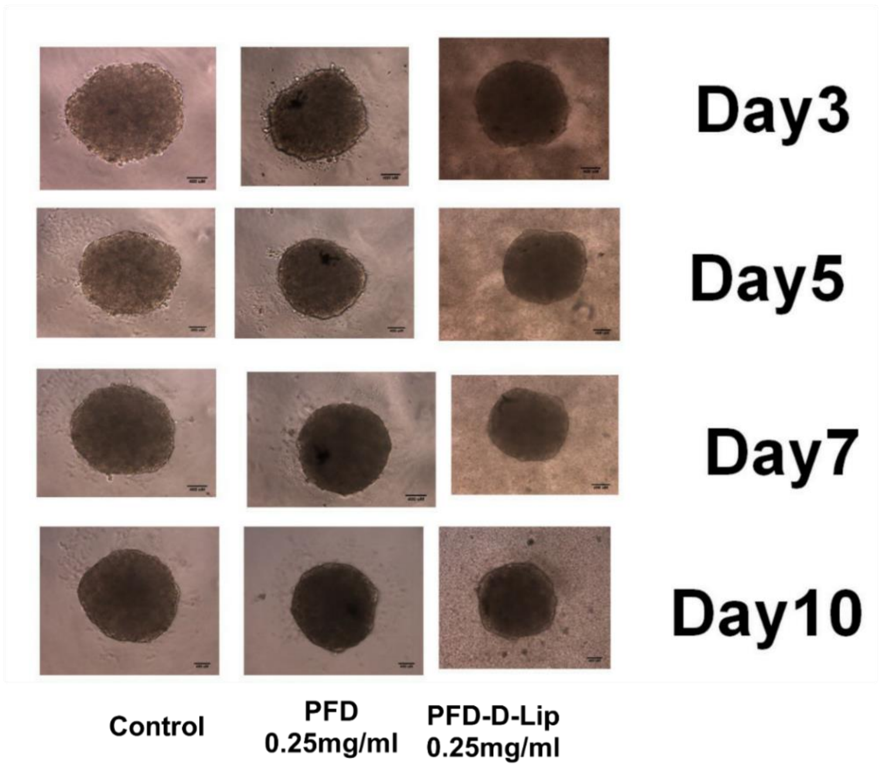

Figure S5. Prophylactic spheroid study: Representing spheroid images on day 3, 5, 7 10 and 15 after treating with control, PFD-Lip and PFD-D-Lip (0.25 mg/mL). A significant reduction was observed in the diameter and volumes of spheroids in presence of PFD-D-Lip as compared to PFD and control. Data represent mean ± SD (*n* = 6).
